# The interventional effect of astragaloside IV on rodent models of myocardial fibrosis: a systematic review and meta-analysis

**DOI:** 10.3389/fphar.2025.1625774

**Published:** 2025-09-22

**Authors:** Haozhe Li, Yunhang Chu, Yue Wang, Yuan Wang, Yebo Zhang

**Affiliations:** ^1^ College of Traditional Chinese Medicine, Inner Mongolia Medical University, Hohhot, China; ^2^ College of Chinese Medicine, Changchun University of Chinese Medicine, Changchun, China; ^3^ Inner Mongolia Clinical Medical College, Inner Mongolia Medical University, Hohhot, China; ^4^ Inner Mongolia Autonomous Region People’s Hospital, Department of Traditional Chinese Medicine, Hohhot, China

**Keywords:** astragaloside IV, myocardial fibrosis, rodent, systematic review, meta-analysis

## Abstract

**Objective:**

This study was conducted to evaluate the interventional effects of astragaloside in a rodent model of myocardial fibrosis (MF).

**Methods:**

Data from studies related to the intervention of astragaloside IV (AS-IV) in rodent models with myocardial fibrosis were systematically retrieved and extracted. The outcome indices included collagen volume fraction (CVF), left ventricular end-systolic diameter (LVESd), left ventricular end-diastolic diameter (LVEDd), interventricular septal thickness at diastole (IVSd), left ventricular posterior wall diastolic thickness (LVPWd), left ventricular internal diameter at diastole (LVIDd), left ventricular mass index (LVMI), left ventricular fractional shortening (LVFS), left ventricular end-diastolic pressure (LVEDp), left ventricular systolic pressure (LVSP), left ventricular internal diameter at systole (LVIDs), left ventricular ejection fraction (LVEF), maximum rate of systolic pressure rise (+dp/dtmax), maximum rate of diastolic pressure fall (−dp/dtmax), and other hemodynamic indices. Additionally, it included lactate dehydrogenase (LDH), tumor necrosis factor-alpha (TNF-α), body weight (BW), and heart rate (HR). The methodological quality of the studies was assessed using the SYRCLE risk of bias tool, and these results were statistically analyzed by meta-analysis. Additionally, meta-regression and subgroup analyses were performed according to species, administration dosage, and administration duration, aiming to further deepen the understanding of the study results and provide references for relevant clinical research.

**Results:**

A total of 38 studies were incorporated into the meta-analysis. The findings indicated that AS-IV led to a reduction in morphostructural indices, including CVF, LVESd, LVEDd, IVSd, LVPWd, and LVMI. Moreover, it decreased LVEDp and LVSP, while increasing hemodynamic indices such as LVEF, LVFS, +dp/dtmax, and −dp/dtmax. Additionally, astragaloside decreased biochemical and physiological indices, including LDH, TNF-α, HR, and BW. However, it exerted no significant impact on the levels of LVIDs and LVIDd in the model.

**Conclusion:**

AS-IV can be used as a supportive treatment for MF, acting through various mechanisms, including the relief of inflammation, myocardial injury, and oxidative stress, thereby contributing to the improvement of ventricular diastolic and contractile capacity and reducing the necrosis and apoptosis of cardiomyocytes.

**Systematic Review:**

https://www.crd.york.ac.uk/PROSPERO/myprospero, identifer CRD420250637182.

## 1 Introduction

Cardiovascular diseases are the primary determinants of global incidence and mortality rates. In 2020 alone, close to one million fatalities attributable to cardiovascular diseases occurred in the United States (Minhas et al., 2024). The prevalence and mortality rates of cardiovascular diseases in China are still on the rise (Majmundar et al., 2023). Based on the statistics and inferences drawn from relevant research data, the number of patients currently afflicted with cardiovascular diseases in China is estimated to be approximately 300 million (Wang et al., 2023). Myocardial fibrosis (MF) is an important pathological process in cardiovascular diseases. It is a pathological change caused by the excessive accumulation of collagen fibers in normal myocardial tissues due to various reasons, which leads to a significant increase in collagen concentration and collagen volume fraction (Wang et al., 2025). Notably, excessive myocardial fibrosis predisposes the patient to cardiac diastolic dysfunction (Barton et al., 2023), thereby inducing arrhythmia, promoting cardiac remodeling and vascular structural alterations, and exacerbating cardiovascular mortality and recurrence.

**FIGURE 1:**
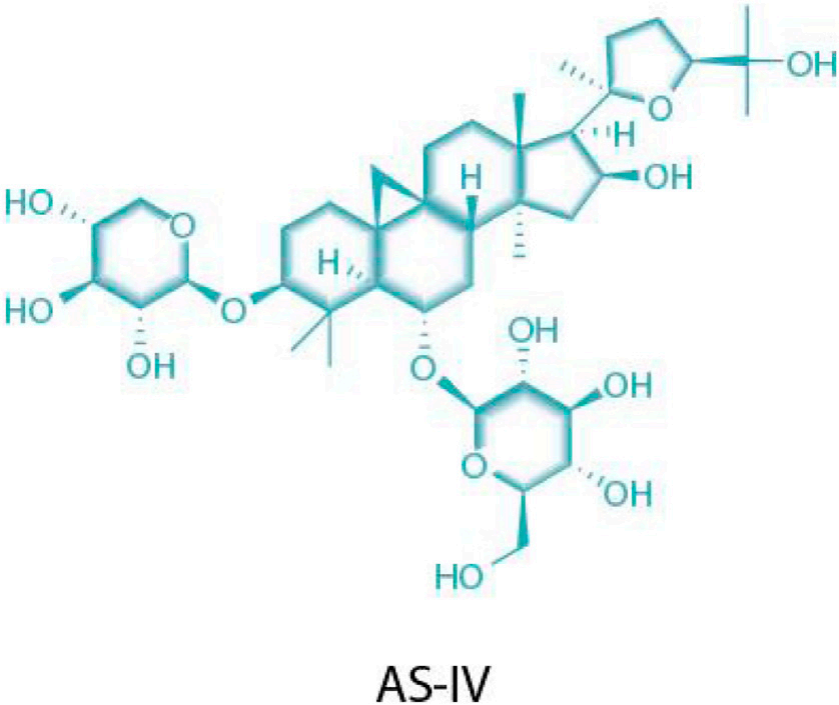
Structures of AS-IV.

AS-IV is an important bioactive component extracted from *Astragalus membranaceus* (a leguminous plant), belonging to the class of tetracyclic triterpenoid saponins. Its chemical structure consists of two parts: one is the aglycone moiety (a triterpenoid, such as cycloastragenol), which serves as the structural core; the other is the glycosyl moiety, which undergoes catalytic modification by glycosyltransferases to form saponin molecules with diverse structures (M et al., 2023) (Figure 1). Extensive pharmacological effects of AS-IV have been documented in recent studies (Li et al., 2023a), with accumulating evidence supporting its multi-targeted pharmacological activities and significant therapeutic potential in cardiovascular diseases. Specifically in the context of MF, AS-IV exerts protective effects through multidimensional regulatory mechanisms, including: 1) modulating pro-inflammatory cytokines; 2) alleviating oxidative stress; 3) inhibiting cardiomyocyte apoptosis; 4) improving ischemia–reperfusion injury; 5) regulating TRPM7 channels and TGF-β1/Smad/NF-κB signaling; and 6) activating the AKT/GSK3-β/SNAIL pathway to counteract epithelial–mesenchymal transition (EMT) (Wang Q. et al., 2021; Yao et al., 2023; Fu et al., 2020; Rios et al., 2022).

This study aims to systematically evaluate the therapeutic effects of AS-IV on MF in rodent models by integrating existing experimental data through a meta-analysis. By elucidating the core mechanisms underlying its anti-fibrotic actions, this research seeks to provide evidence-based support for translating AS-IV from preclinical studies to clinical applications. Specifically, the findings may facilitate the development of novel MF-targeted therapies and offer innovative perspectives for integrative Chinese–Western medicine approaches in cardiovascular disease management.

## 2 Materials and methods

### 2.1 Search strategy

A systematic search was conducted across the PubMed, Web of Science, Embase, Cochrane Library, CNKI, Wanfang Data, and VIP databases for studies investigating AS-IV intervention in rodent models of myocardial fibrosis. The search spanned from each database’s inception through 24 November 2024. Key search terms included: 1) Astragaloside IV/AS-IV; 2) myocardial fibrosis; 3) rats/mice. Terms were combined using logical “AND” operators. The specific search formula is provided in Supplementary Material 1.

### 2.2 Inclusion criteria

1) Study subjects: Rodent models with MF confirmed by indicator detection.
2) Study design: Controlled animal experiments involving MF modeling methods, with experimental animals of any species or strain, and no language restrictions applied to the included literature.
3) Intervention measures: Administration of AS-IV or its preparations was required. Negative control groups received no medication or placebo.
4) Outcome indicators: To ensure key parameters could be directly extracted or derived through calculation, drug efficacy metrics were required to be presented numerically. Measured indices included collagen volume fraction (CVF), left ventricular end-systolic diameter (LVESd), left ventricular end-diastolic diameter (LVEDd), interventricular septal thickness at diastole (IVSd), left ventricular posterior wall diastolic thickness (LVPWd), left ventricular internal diameter at diastole (LVIDd), left ventricular mass index (LVMI), left ventricular fractional shortening (LVFS), left ventricular end-diastolic pressure (LVEDp), left ventricular systolic pressure (LVSP), left ventricular internal diameter at systole (LVIDs), left ventricular ejection fraction (LVEF), maximum rate of systolic pressure rise (+dp/dtmax), maximum rate of diastolic pressure fall (−dp/dtmax), and other hemodynamic indices. Additionally, it included lactate dehydrogenase (LDH), tumor necrosis factor-alpha (TNF-α), body weight (BW), and heart rate (HR). These parameters included both mean values and standard deviations.

### 2.3 Exclusion criteria

1) The data for evaluation indicators were incomplete.
2) The article comprises systematic reviews, meta-analyses, and *in vitro* experimental studies.
3) Experimental groups or control groups involved the administration of drugs other than AS-IV.
4) The experimental animals in the study were not rodents.

### 2.4 Literature screening and data extraction

According to the method of including studies in version 5.0.2 of the Cochrane Collaboration’s Handbook for Systematic Reviewers, the retrieval results from each database were imported into the literature management software Zotero. In parallel and independently, two reviewers, Li Haozhe and Chu Yunhang, screened the literature and then extracted the data and cross-checked the experimental results. In case of disagreement, they reached a decision through negotiation or through referring the matter to Wang Yue for judgment. Finally, the eligible literature data were classified and statistically analyzed using MS Excel. The data to be extracted included author information, publication year of the literature, drugs used in the experiment, specific modeling methods, number of models, route of drug administration, numerical value of the drug dosage, categories of detection indicators, corresponding units of the detection indicators, and final experimental result data. To ensure the validity of the data and the reliability of the analysis, when the number of studies related to a certain outcome indicator was less than 3, this outcome indicator would be excluded from the scope of the study.

Additionally, this systematic review was registered in the PROSPERO International Prospective Register of Systematic Reviews (registration number: CRD420250637182) to help avoid duplication and reduce the possibility of reporting bias by comparing the completed evaluation with the planned protocol.

### 2.5 Statistical methods

Meta-analyses were performed using RevMan 5.1 software. For categorical data, the risk ratio (RR) was used as the effect size measure. For continuous data, the mean difference (MD) was employed. When outcome measures shared identical units and methods, the weighted mean difference (WMD) was calculated; otherwise, the standardized mean difference (SMD) was used. All effect sizes were reported with 95% confidence intervals (CI). Heterogeneity among included studies was evaluated using Cochran’s Q test (significance level: *P* < 0.1) and quantified by the I^2^ statistic. A fixed-effect model was applied when I^2^ ≤ 50% indicated low heterogeneity, while a random-effect model was used for high heterogeneity (I^2^ > 50%), followed by exploration of heterogeneity sources. Publication bias was initially assessed visually via funnel plots. Additionally, StataSE 12.0 software was used to conduct a leave-one-out method to investigate potential heterogeneity sources, complemented by Egger’s test for publication bias detection. Subgroup analyses and meta-regression analyses were performed to identify the root causes of significant heterogeneity.

## 3 Results

### 3.1 Results of the literature review

**FIGURE 2:**
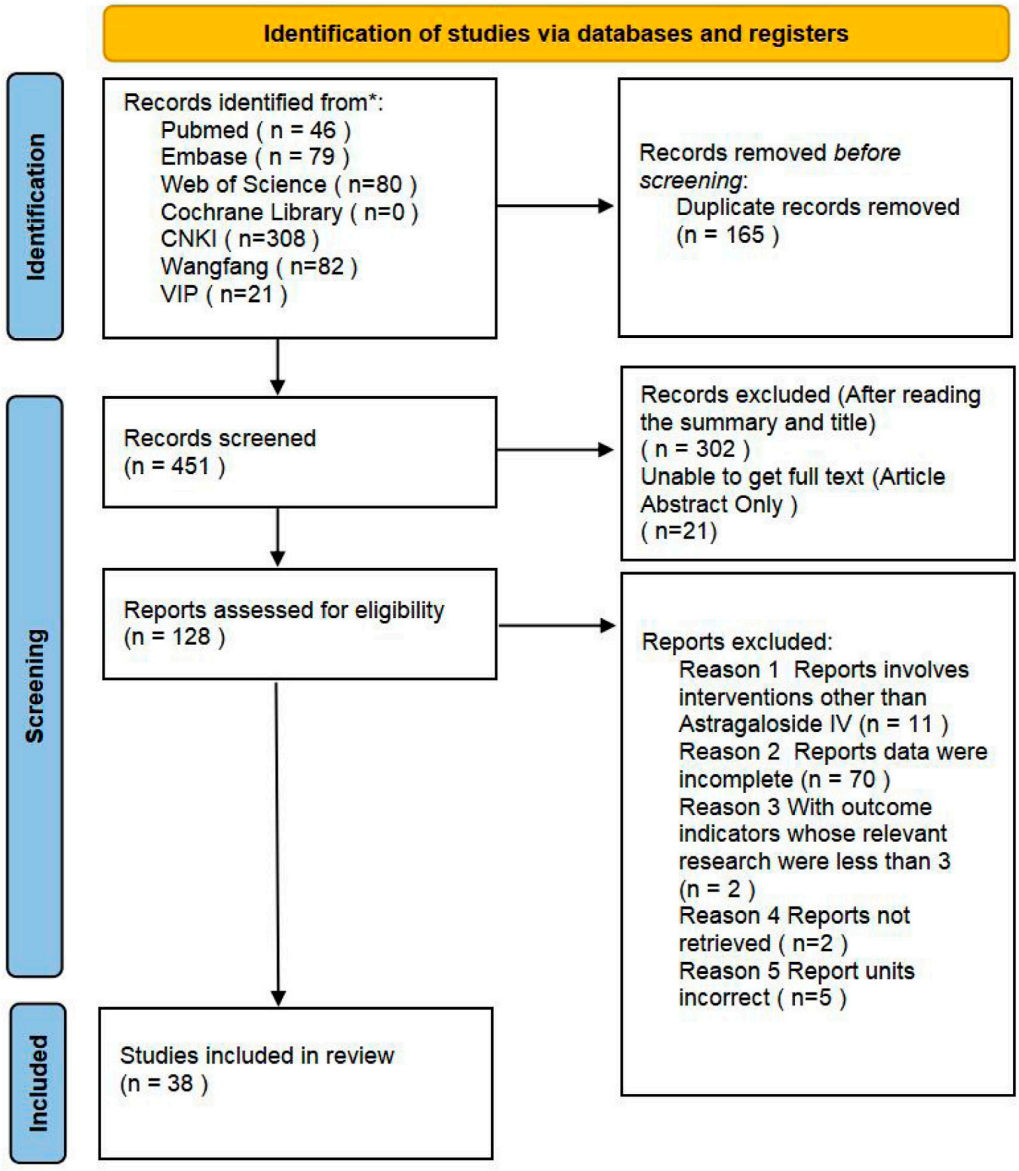
Flowchart of the eligible literature search process.

The literature retrieval process for this meta-analysis is outlined in Figure 2. A total of 616 relevant articles were identified through searches in databases including PubMed. After systematic screening, 38 eligible studies see Table 1 for details. (Rios et al., 2022; Chen and Wang, 2017; Fathiazad et al., 2019; Jiang and Zhang, 2016; Jiang and Wei, 2016; Jiang, 2016; Lin et al., 2019; Luo et al., 2020; Lv, 2018; Liu et al., 2022; Li et al., 2005; Li, 2021; Chen et al., 2011; Tang et al., 2016; Tang, 2017; Tang et al., 2017a; Liu et al., 2019; Wu et al., 2023; Wang SF. et al., 2020; Zhang et al., 2022; Xue et al., 2024; Xu et al., 2010; Zhao, 2020; Zhang et al., 2007; Zhang, 2021; Wang Z. et al., 2020; Cheng, 2017; Gong and Ke, 2024; He, 2013; Li et al., 2017; Li, 2013; Tang et al., 2017b; Wang et al., 2024; Wang XL. et al., 2021; Yan et al., 2024; Zhang, 2012; Zhang, 2015; Zhang et al., 2023) were included in the final analysis. Data extraction included study-specific identifiers, experimental subjects, modeling methods, and other study characteristics. Among the included studies, those with three groups typically consisted of a sham operation group, a model group, and an astragaloside IV treatment group. Studies with five groups additionally incorporated three subgroups receiving different doses of AS-IV, in addition to the sham and model groups.

**TABLE 1:** Key characteristics of included studies.

Study inclusion	Animal species	Dosage	Treatment/control	Administration route	Treatment duration (days)	Modeling method	Outcome
CQ 2017 (Chen and Wang, 2017)	Sprague–Dawley (SD) rat	AS-IV 20 mg/kg, 40 mg/kg, and 80 mg/kg	30/10	Oral gavage	14	ISO-induced model	② LVEF, ⑤ LVFS, ⑩ CVF, ⑰ BW
FF 2019 (Fathiazad et al., 2019)	Male Wistar rats	AS-IV 2.5 mg/kg, 5 mg/kg, and 10 mg/kg	18/6	Oral administration	4	ISO-induced model	⑱ HR
JHQ 2016.1 (Jiang and Zhang, 2016)	Male Sprague–Dawley rats	AS-IV 20 mg/kg, 40 mg/kg, and 60 mg/kg	36/12	Oral gavage	28	Abdominal aortic constriction model	⑥ LVEDp, ⑩ CVF, ⑪ LVSP, ⑮ +dp/dtmax, ⑯ −dp/dtmax
JHQ 2016.2 (Jiang and Wei, 2016)	Male Sprague–Dawley rats	AS-IV 20 mg/kg, 40 mg/kg, and 80 mg/kg	36/12	Oral gavage	28	Abdominal aortic constriction model	⑥ LVEDp, ⑩ CVF, ⑪ LVSP, ⑮ +dp/dtmax, ⑯ −dp/dtmax
JHQ 2017 (Jiang, 2016)	Male Sprague–Dawley rats	AS-IV 20 mg/kg, 40 mg/kg, and 60 mg/kg	39/13	Oral gavage	28	Abdominal aortic constriction model	① LVESd, ③ LVEDd, ⑥ LVEDp, ⑪ LVSP, ⑮ +dp/dtmax, ⑯ −dp/dtmax
JML 2019 (Lin et al., 2019)	C57BL/6J mouse	AS-IV 40 mg/kg	20/20	Oral administration	28	DOX IP-induced model	② LVEF, ⑤ LVFS, ⑧ IVSd, ⑨ LVPWd, ⑫ LDH, ⑭ LVIDd
LQ 2020 (Luo et al., 2020)	Sprague–Dawley (SD) rat	AS-IV 20 mg/kg, 40 mg/kg, and 80 mg/kg	39/13	Oral gavage	56	Left atrial appendage root ligation model	① LVESd, ② LVEF, ③ LVEDd, ④ LVMI, ⑤ LVFS
LQW 2018 (Lv, 2018)	Male Sprague–Dawley rats	AS-IV 30 mg/kg and 70 mg/kg	40/20	-	56	Abdominal aortic constriction model	① LVESd, ③ LVEDd, ④ LVMI, ⑥ LVEDp, ⑨ LVPWd, ⑩ CVF, ⑪ LVSP
LY 2022 (Liu et al., 2022)	Male Wistar rats	AS-IV 6.9 mg/kg	5/5	Oral gavage	56	HFD/STZ-induced model	① LVESd, ② LVEF, ③ LVEDd, ⑤ LVFS
LZP 2005 (Li et al., 2005)	Male Wistar rats	AS-IV 2.5 mg/kg, 5.0 mg/kg, 10 mg/kg, 15 mg/kg, and 20 mg/kg	40/10	Intraperitoneal injection	28	LAD ligation model	⑱ HR
LMH 2021 (Li, 2021)	Male Sprague–Dawley rats	AS-IV 40 mg/kg	8/8	Intravenous injection	28	LAD ligation model	② LVEF, ⑤ LVFS, ⑬ LVIDs, ⑭ LVIDd
PC 2011 (Chen et al., 2011)	BALB/c mouse	AS-IV 0.6 mg/kg	20/30	Oral administration	90	CVB3-induced model via IP injection	② LVEF, ③ LVEDd, ⑤ LVFS
TB 2016 (Tang et al., 2016)	Male Sprague–Dawley rats	AS-IV 30 mg/kg and 70 mg/kg	40/20	Oral gavage	56	Abdominal aortic constriction model	① LVESd, ③ LVEDd, ④ LVMI, ⑥ LVEDp, ⑨ LVPWd, ⑩ CVF, ⑪ LVSP, ⑮ +dp/dtmax, ⑯ −dp/dtmax, ⑰ BW
TB 2017.2 (Tang, 2017)	Male Sprague–Dawley rats	AS-IV 25 mg/kg and 50 mg/kg	44/22	Oral gavage	56	Abdominal aortic constriction model	① LVESd, ② LVEF, ③ LVEDd, ⑥ LVEDp, ⑨ LVPWd, ⑮ +dp/dtmax, ⑯ −dp/dtmax, ⑰ BW
TB 2017.1 (Tang et al., 2017a)	Male Sprague-Dawley rats	AS-IV 30 mg/kg	20/20	Oral gavage	56	Abdominal aortic constriction model	① LVESd, ③ LVEDd, ⑥ LVEDp, ⑨ LVPWd, ⑪ LVSP, ⑮ +dp/dtmax, ⑯ −dp/dtmax
TLL 2019 (Liu et al., 2019)	C57BL/6J mouse	AS-IV 100 mg/kg	20/20	Oral gavage	14	CVB3-induced model via IP injection	② LVEF, ⑨ LVPWd
WFF 2023 (Wu et al., 2023)	C57BL/6J mouse	AS-IV 100 mg/kg	15/15	Intraperitoneal injection	30	ISO-induced model	② LVEF, ⑫ LDH
WSF 2020 (Wang et al., 2020a)	Male Sprague–Dawley rats	AS-IV 2 mg/kg	20/20	Oral gavage	56	Left atrial appendage root ligation model	② LVEF, ⑤ LVFS, ⑦ TNF-α
WZ 2022 (Zhang et al., 2022)	C57BL/6J mouse	AS-IV 10 mg/kg	10/10	Intraperitoneal injection	28	LAD ligation model	② LVEF, ⑤ LVFS, ⑧ IVSd, ⑬ LVIDs, ⑭ LVIDd
XSY 2024 (Xue et al., 2024)	XXL2010	AS-IV 20 mg/kg, 40 mg/kg, and 80 mg/kg	30/10	Oral gavage	42	DOX IP-induced model	① LVESd, ② LVEF, ③ LVEDd, ⑦ TNF-α
XXL 2010 (Xu et al., 2010)	Male Kunming mice	AS-IV 40 mg/kg and 80 mg/kg	16/8	Oral gavage	14	ISO-induced model	② LVEF, ⑤ LVFS, ⑬ LVIDs, ⑭ LVIDd
ZK 2020 (Zhao, 2020)	Male Sprague–Dawley rats	AS-IV 40 mg/kg and 80 mg/kg	16/8	Oral gavage	63	Abdominal aortic constriction model	② LVEF, ④ LVMI, ⑧ IVSd, ⑨ LVPWd
ZSC 2007 (Zhang et al., 2007)	BALB/c mouse	AS-IV 0.6 mg/kg	30/30	Oral administration	90	CVB3-induced model via IP injection	⑩ CVF
ZXX 2021 (Zhang, 2021)	Male Dahl SS rats	AS-IV 10 mg/kg, 20 mg/kg, and 40 mg/kg	15/5	Oral gavage	56	High-salt diet-induced model	② LVEF, ⑧ IVSd, ⑨ LVPWd, ⑭ LVIDd, ⑱ HR
ZYW 2020 (Wang et al., 2020b)	Male Sprague–Dawley rats	AS-IV 80 mg/kg and 200 mg/kg	10/5	Oral gavage	56	HFD/STZ-induced model	⑥ LVEDp, ⑪ LVSP, ⑮ +dp/dtmax, ⑯ −dp/dtmax
CJ 2017 (Cheng, 2017)	Male Sprague–Dawley rats	AS-IV 30 mg/kg and 70 mg/kg	20/10	Oral gavage	56	LAD ligation model	⑥ LVEDp, ⑦ TNF-α, ⑪ LVSP, ⑮ +dp/dtmax, ⑯ −dp/dtmax, ⑱ HR
GS 2024 (Gong and Ke, 2024)	Sprague–Dawley rats	AS-IV 10 mg/kg, 20 mg/kg, and 50 mg/kg	30/10	Subcutaneous injection	14	Subcutaneous implantation of AngⅡ slow-release pump	③ LVEDd, ⑤ LVFS
HHY 2013 (He, 2013)	Sprague–Dawley rats	AS-IV 40 mg/kg and 80 mg/kg	16/8	Oral gavage	84	Abdominal aortic constriction model	④ LVMI, ⑦ TNF-α
LMF 2017 (Li et al., 2017)	C57BL/6J mouse	AS-IV 40 mg/kg and 80 mg/kg	12/6	Oral gavage	8 h	Intraperitoneal LPS injection	② LVEF, ⑤ LVFS, ⑦ TNF-α, ⑬ LVIDs, ⑭ LVIDd
LZZ 2013 (Li, 2013)	Male Sprague–Dawley rats	AS-IV 5 mg/kg, 10 mg/kg, and 20 mg/kg	30/10	Intraperitoneal injection	7	ISO-induced model	⑥ LVEDp, ⑪ LVSP, ⑫ LDH
TB 2017.3 (Tang et al., 2017b)	Sprague–Dawley rats	AS-IV 40 mg/kg and 80 mg/kg	38/18	Oral gavage	56	Abdominal aortic constriction model	① LVESd, ③ LVEDd, ⑥ LVEDp, ⑨ LVPWd, ⑩ CVF, ⑪ LVSP, ⑮ +dp/dtmax, ⑯ −dp/dtmax
WLY 2024 (Wang et al., 2024)	Male Sprague–Dawley rats	AS-IV 20 mg/kg, 40 mg/kg, and 80 mg/kg	24/8	Oral gavage	56	LAD ligation model	① LVESd, ② LVEF, ③ LVEDd, ④ LVMI, ⑤ LVFS
WXL 2021 (Wang et al., 2021b)	ICR mice	AS-IV 80 mg/kg	10/10	Oral gavage	10	DOX IP-induced model	② LVEF, ⑤ LVFS, ⑦ TNF-α, ⑫ LDH, ⑱ HR
YJY 2024 (Yan et al., 2024)	Male Sprague–Dawley rats	AS-IV 10 mg/kg, 20 mg/kg, and 40 mg/kg	24/8	Oral gavage	14	LAD ligation model	② LVEF, ⑤ LVFS
YJY 2024.1 (Yan et al., 2024)	Male Sprague–Dawley rats	AS-IV 10 mg/kg, 20 mg/kg, and 40 mg/kg	20/8	Oral gavage	28	LAD ligation model	② LVEF, ⑤ LVFS
ZJ 2012 (Zhang, 2012)	Sprague–Dawley rats	AS-IV 1 mg/kg and 5 mg/kg	30/15	Intraperitoneal injection	84	Abdominal aortic constriction model	④ LVMI, ⑥ LVEDp, ⑪ LVSP, ⑮ +dp/dtmax, ⑯ −dp/dtmax, ⑱ HR
ZSP 2015 (Zhang, 2015)	Male Sprague–Dawley rats	AS-IV 80 mg/kg	10/10	Oral gavage	14	ISO-induced model	④ LVMI
ZYH 2023 (Zhang et al., 2023)	Male Sprague–Dawley rats	AS-IV 20 mg/kg and 40 mg/kg	16/8	Intraperitoneal injection	28	ISO-induced model	② LVEF, ⑤ LVFS, ⑫ LDH
JWW 2023 (Jia et al., 2023)	Male Sprague–Dawley rats	AS-IV 20 mg/kg, 40 mg/kg, and 80 mg/kg	16/8	Intraperitoneal injection	28	ISO-induced model	② LVEF, ⑤ LVFS, ⑫ LDH

① LVESd; ② LVEF; ③ LVEDd; ④ LVMI; ⑤ LVFS; ⑥ LVEDp; ⑦ TNF-α; ⑧ IVSd; ⑨ LVPWd; ⑩ CVF; ⑪ LVSP; ⑫ LDH; ⑬ LVIDs; ⑭ LVIDd; ⑮ +dp/dtmax; ⑯ −dp/dtmax; ⑰ BW; ⑱ HR.

This meta-analysis included 38 designs involving 1,334 rodent models (891 in AS-IV treatment groups vs. 443 in control groups), with species distribution as follows: 25 studies used Sprague–Dawley rats (Rios et al., 2022; Fathiazad et al., 2019; Jiang and Zhang, 2016; Jiang and Wei, 2016; Lin et al., 2019; Luo et al., 2020; Li et al., 2005; Chen et al., 2011; Tang et al., 2016; Tang, 2017; Wu et al., 2023; Zhang et al., 2022; Xu et al., 2010; Zhang, 2021; Wang Z. et al., 2020; Cheng, 2017; Gong and Ke, 2024; Li et al., 2017; Li, 2013; Tang et al., 2017b; Wang XL. et al., 2021; Yan et al., 2024; Zhang, 2012; Zhang, 2015; Zhang et al., 2023), three studies used Wistar rats (Chen and Wang, 2017; Lv, 2018; Liu et al., 2022), one study used Dahl SS rats (Zhang et al., 2007), five studies used C57BL/6J mice (Jiang, 2016; Tang et al., 2017a; Liu et al., 2019; Wang SF. et al., 2020; He, 2013), two studies used BALB/c mice (Li, 2021; Zhao, 2020), and one study each used Kunming mice (Xue et al., 2024) and ICR mice (Wang et al., 2024).

Quality assessment results: The included studies were evaluated using SYRCLE’s Risk of Bias tool for animal experiments. Among all included studies, 25 clearly stated that they used the random number table method to generate allocation sequences (Chen and Wang, 2017; Fathiazad et al., 2019; Jiang and Zhang, 2016; Jiang, 2016; Lin et al., 2019; Luo et al., 2020; Lv, 2018; Liu et al., 2022; Li et al., 2005; Chen et al., 2011; Tang et al., 2016; Tang, 2017; Tang et al., 2017a; Liu et al., 2019; Wu et al., 2023; Wang SF. et al., 2020; Zhang et al., 2022; Xue et al., 2024; Xu et al., 2010; Zhao, 2020; Zhang et al., 2007; Zhang, 2021; Wang Z. et al., 2020; Gong and Ke, 2024; He, 2013; Li et al., 2017; Li, 2013; Tang et al., 2017b; Wang et al., 2024; Wang XL. et al., 2021; Zhang, 2012; Zhang, 2015; Zhang et al., 2023; Jia et al., 2023), and two did not adopt this method for determining allocation sequences (Jiang and Wei, 2016; Li, 2021). Studies that did not adopt the random number table method may introduce a certain degree of subjectivity into the grouping process, making it difficult to ensure fairness and comparability between groups, thereby affecting the reliability of the research results. Two studies provided no clear description of how they allocated subjects (Cheng, 2017; Yan et al., 2024), and the scientificity and rationality of their allocation sequence generation are questionable, which increases the risk of bias. However, none of the studies mentioned allocation concealment, random housing of animals, or blinding of relevant personnel and outcome assessors. The absence of these key links may introduce biases throughout the process from grouping and feeding to outcome assessment, ultimately undermining the credibility of the experimental conclusions regarding astragaloside IV. All included animals were incorporated into the final analysis, with no instances of selectively choosing animals for evaluation. Although three studies contained incomplete data (Chen and Wang, 2017; Lin et al., 2019; Liu et al., 2019), these gaps were accurately explained and reasonably justified, confirming the completeness of reporting and ruling out any association with selective reporting see Table 2 for details.

**TABLE 2:** Risk of bias assessment of included studies.

Study inclusion	1	2	3	4	5	6	7	8	9	10
CQ2017 (Chen and Wang, 2017)	Y	Y	U	U	U	N	U	Y	N	U
FF2019 (Fathiazad et al., 2019)	Y	Y	U	U	U	N	U	N	N	U
JHQ2016.1 (Jiang and Zhang, 2016)	Y	Y	U	U	U	N	U	N	N	U
JHQ2016.2 (Jiang and Wei, 2016)	N	Y	U	U	U	N	U	N	N	U
JHQ2017 (Jiang, 2016)	Y	Y	U	U	U	N	U	N	N	U
JML2019 (Lin et al., 2019)	Y	Y	U	U	U	N	U	Y	N	U
LQ2020 (Luo et al., 2020)	Y	Y	U	U	U	N	U	N	N	U
LQW2018 (Lv, 2018)	Y	Y	U	U	U	N	U	N	N	U
LY2022 (Liu et al., 2022)	Y	Y	U	U	U	N	U	N	N	U
LZP2005 (Li et al., 2005)	Y	Y	U	U	U	N	U	N	N	U
LMH2021 (Li, 2021)	N	Y	U	U	U	N	U	N	N	U
PC2011 (Chen et al., 2011)	Y	Y	U	U	U	N	U	N	N	U
TB2016 (Tang et al., 2016)	Y	Y	U	U	U	N	U	N	N	U
TB2017.2 (Tang, 2017)	Y	Y	U	U	U	N	U	N	N	U
TB2017.1 (Tang et al., 2017a)	Y	Y	U	U	U	N	U	N	N	U
TLL2019 (Liu et al., 2019)	Y	Y	U	U	U	N	U	Y	N	U
WFF2023 (Wu et al., 2023)	Y	Y	U	U	U	N	U	N	N	U
WSF2020 (Wang et al., 2020a)	Y	Y	U	U	U	N	U	N	N	U
WZ2022 (Zhang et al., 2022)	Y	Y	U	U	U	N	U	N	N	U
XSY2024 (Xue et al., 2024)	Y	Y	U	U	U	N	U	N	N	U
XXL2010 (Xu et al., 2010)	Y	Y	U	U	U	N	U	N	N	U
ZK2020 (Zhao, 2020)	Y	Y	U	U	U	N	U	N	N	U
ZSC2007 (Zhang et al., 2007)	Y	Y	U	U	U	N	U	N	N	U
ZXX2021 (Zhang, 2021)	Y	Y	U	U	U	N	U	N	N	U
ZYW2020 (Wang et al., 2020b)	Y	Y	U	U	U	N	U	N	N	U
CJ2017 (Cheng, 2017)	U	Y	U	U	U	N	U	N	N	U
GS2024 (Gong and Ke, 2024)	Y	Y	U	U	U	N	U	N	N	U
HHY2013 (He, 2013)	Y	Y	U	U	U	N	U	N	N	U
LMF2017 (Li et al., 2017)	Y	Y	U	U	U	N	U	N	N	U
LZZ2013 (Li, 2013)	Y	Y	U	U	U	N	U	N	N	U
TB2017.3 (Tang et al., 2017b)	Y	Y	U	U	U	N	U	N	N	U
WLY2024 (Wang et al., 2024)	Y	Y	U	U	U	N	U	N	N	U
WXL2021 (Wang et al., 2021b)	Y	Y	U	U	U	N	U	N	N	U
YJY2024 (Yan et al., 2024)	U	Y	U	U	U	N	U	N	N	U
YJY2024.1 (Yan et al., 2024)	U	Y	U	U	U	N	U	N	N	U
ZJ2012 (Zhang, 2012)	Y	Y	U	U	U	N	U	N	N	U
ZSP2015 (Zhang, 2015)	Y	Y	U	U	U	N	U	N	N	U
ZYH2023 (Zhang et al., 2023)	Y	Y	U	U	U	N	U	N	N	U
JWW2023 (Jia et al., 2023)	Y	Y	U	U	U	N	U	N	N	U

1 Random sequence generation; 2 Baseline comparability; 3 Allocation concealment; 4 Random housing of animals; 5 Blinding of caregivers/researchers; 6 Random selection for outcome assessment; 7 Blinding of outcome assessors; 8 Incomplete outcome data; 9 Selective outcome reporting; 10 Other sources of bias; Y, yes; N, no; U, unclear.

### 3.2 Meta-analysis results

AS-IV significantly influenced cardiac morphological parameters (CVF, LVESd, LVEDd, IVSd, LVPWd, and LVMI), hemodynamic indices (LVFS, LVEDp, LVSP, LVEF, and ±dp/dtmax), and biochemical/physiological indicators (LDH, TNF-α, BW, and HR) in rodent models of cardiac disease. However, it exerted no significant statistical effect on LVIDd and LVIDs.

#### 3.2.1 Effects of astragaloside IV on cardiac morphological parameters in rodent myocardial fibrosis models

**FIGURE 3:**
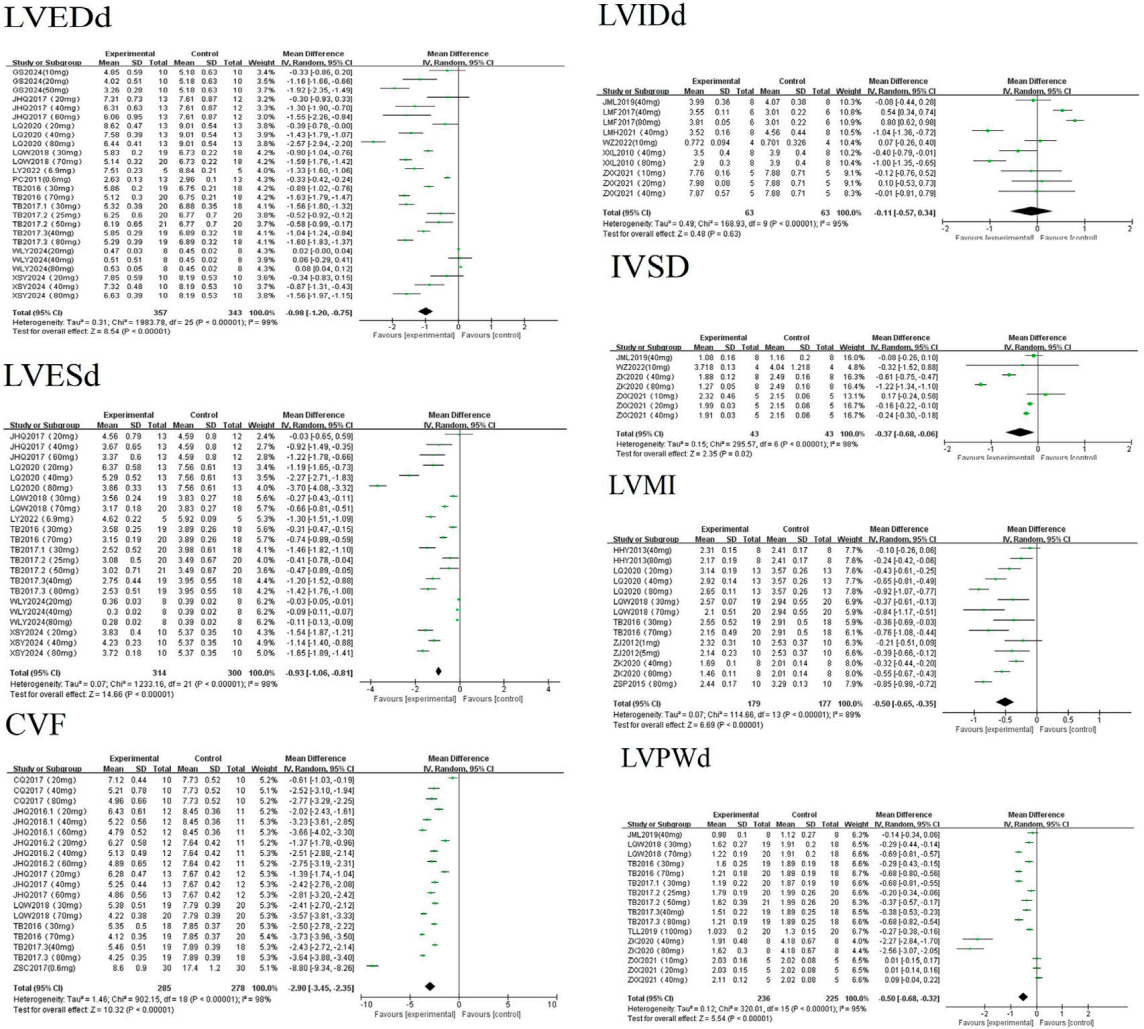
Forest plots of morphostructural parameters.

A random-effects meta-analysis revealed that AS-IV significantly reduced cardiac morphological parameters in experimental models of MF, including CVF [MD = −2.90, 95%CI (−3.45, −2.35), *P* < 0.01], LVESd [MD = −0.93, 95%CI (−1.06, −0.81), *P* < 0.01], LVEDd [MD = −0.98, 95%CI (−1.20, −0.75), *P* < 0.01], IVSd [MD = −0.37, 95%CI (−0.68, −0.06), *P* = 0.02], LVPWd [MD = −0.50, 95%CI (−0.68, −0.32), *P* < 0.01], and LVMI [MD = −0.50, 95%CI (−0.65, −0.35), *P* < 0.01]. Statistically significant reductions were observed for all parameters except LVIDd [MD = −0.11, 95%CI (−0.57, 0.34), *P* = 0.63] see Figure 3. LVIDd, an indicator reflecting ventricular size, has relatively stable values. In clinical practice, patients with myocardial fibrosis often undergo a long-term pathological process; thus, significant changes in LVIDd may be difficult to observe in short-term animal experiments. Inherent variations in pathological phenotypes induced by different modeling approaches lead to significant heterogeneity in baseline LVIDd levels and sensitivity to astragaloside IV. Additionally, the small sample size and systematic errors introduced by operators during ultrasonic measurements might have masked the potential effects of AS-IV.

#### 3.2.2 Effects of astragaloside IV on hemodynamic parameters in rodent myocardial fibrosis models

**FIGURE 4:**
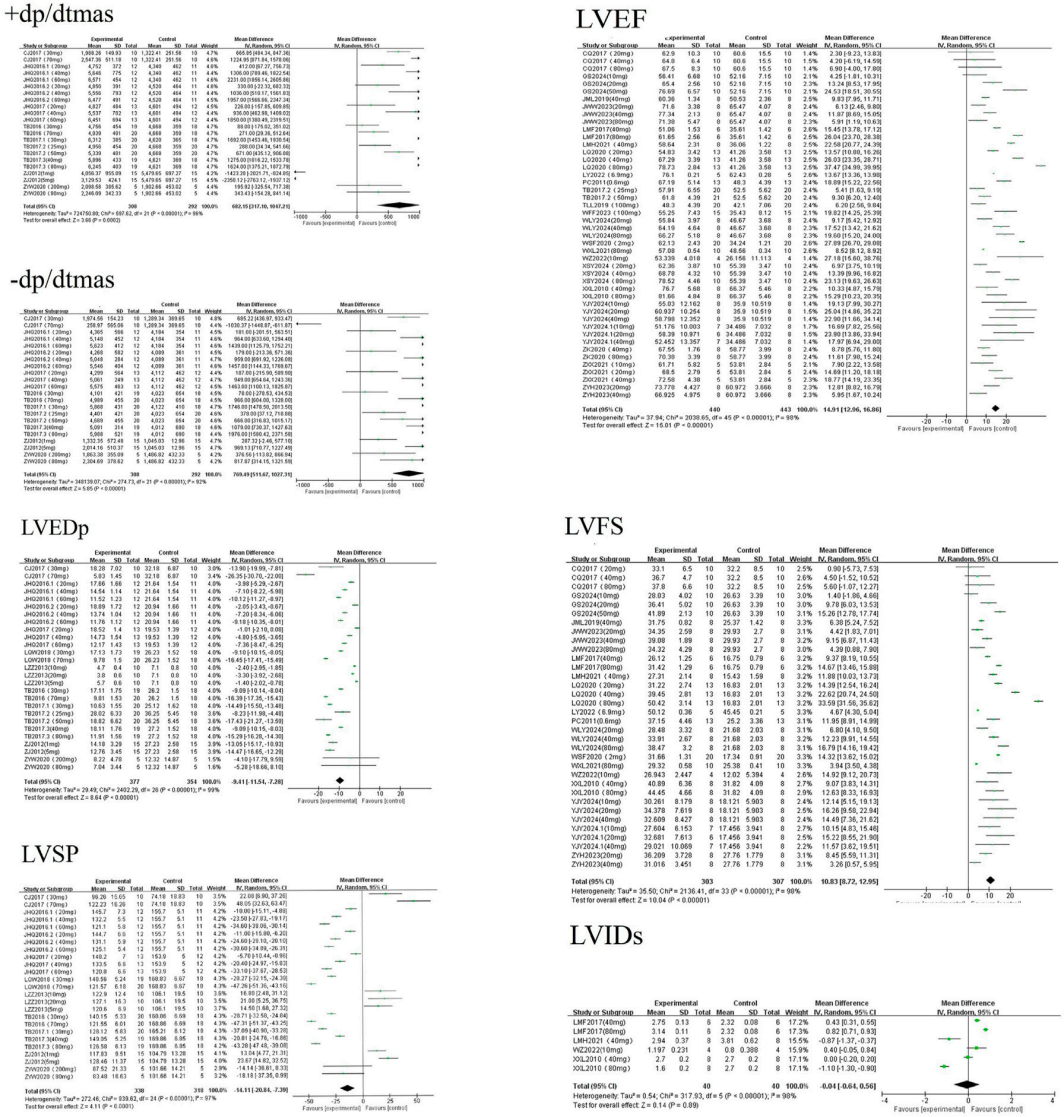
Forest plots of hemodynamic parameters.

A random-effects meta-analysis revealed that AS-IV significantly improved cardiac hemodynamic parameters in experimental models of MF, including LVFS [MD = 10.83, 95%CI (8.72, 12.95), *P* < 0.01], LVEF [MD = 14.91, 95%CI (12.96, 16.86), *P* < 0.01], +dp/dtmax [MD = 682.15, 95%CI (317.10, 1047.21), *P* < 0.01], and −dp/dtmax [MD = 769.49, 95%CI (511.67, 1027.31), *P* < 0.01]. Conversely, AS-IV significantly reduced LVEDp [MD = −9.41, 95%CI (−11.54, −7.28), *P* < 0.01] and LVSP [MD = −14.11, 95%CI (−20.84, −7.39), *P* < 0.01]. No statistically significant effect was observed on LVIDs [MD = −0.04, 95%CI (−0.64, 0.56), *P* = 0.07] see Figure 4. The reasons for this lack of statistical significance are presumably similar to those for LVIDd discussed in Section 3.2.1: LVIDs, like LVIDd, is an indicator reflecting ventricular size and has relatively stable values. In clinical practice, patients with myocardial fibrosis often undergo a long-term pathological process; thus, significant changes in LVIDs may be difficult to observe in short-term animal experiments. Moreover, inherent variations in pathological phenotypes induced by different modeling approaches lead to significant heterogeneity in baseline LVIDs levels and sensitivity to AS-IV. Additionally, the small sample size and systematic errors introduced by operators during ultrasonic measurements might have masked the potential effects of AS-IV.

#### 3.2.3 Effects of astragaloside IV on biochemical and physiological parameters in rodent myocardial fibrosis models

**FIGURE 5:**
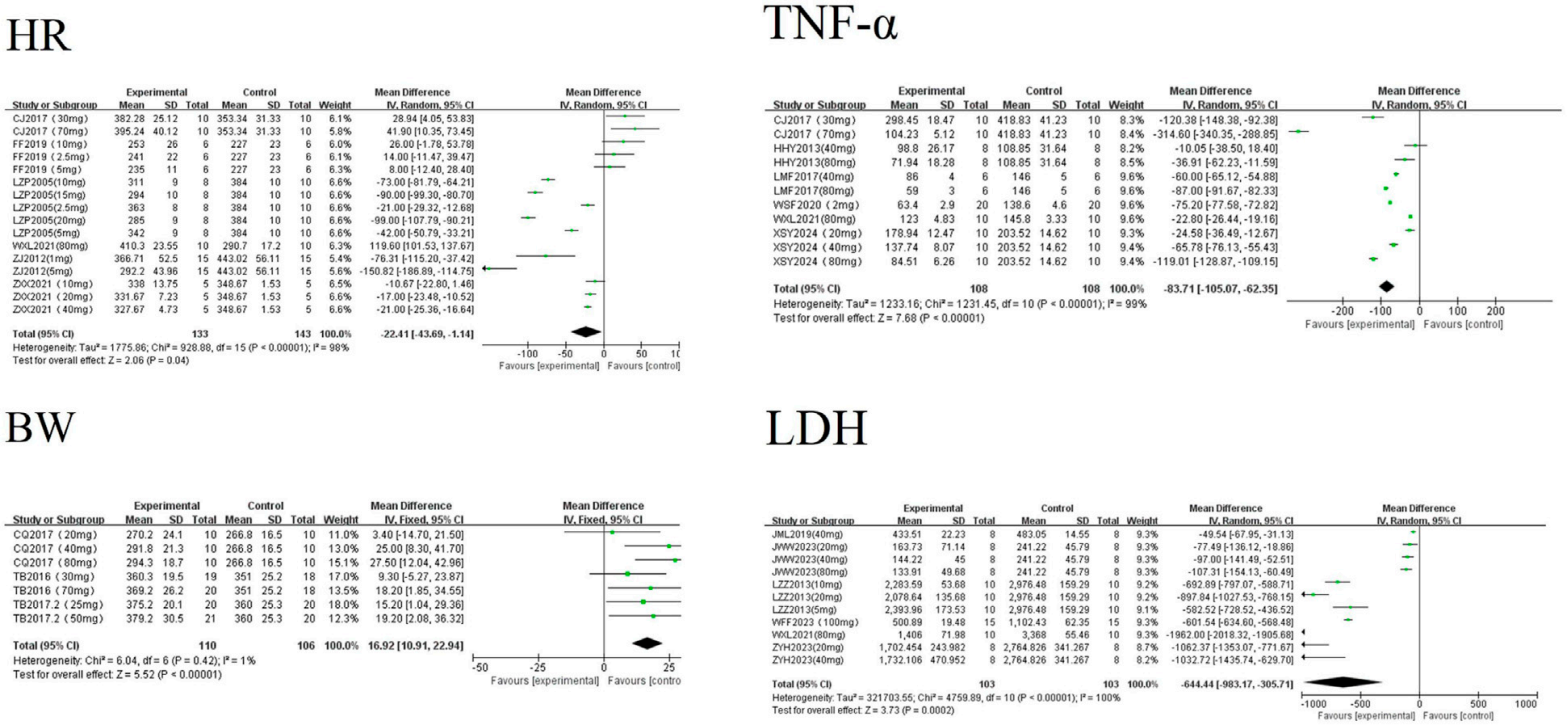
Forest plots related to hemodynamics.

A random-effects meta-analysis revealed that AS-IV significantly reduced levels of LDH [MD = −644.44, 95%CI (−983.17, −305.71), *P* < 0.01], TNF-α [MD = −83.71, 95%CI (−105.07, −62.35), *P* < 0.01], and HR [MD = −22.41, 95%CI (−43.69, −1.14), *P* = 0.04] in experimental models of MF. Conversely, AS-IV significantly increased BW [MD = 16.92, 95%CI (10.91, 22.94), *P* < 0.01] see Figure 5. All observed differences reached statistical significance (*P* < 0.05).

#### 3.2.4 Sensitivity analysis and publication bias

**FIGURE 6:**
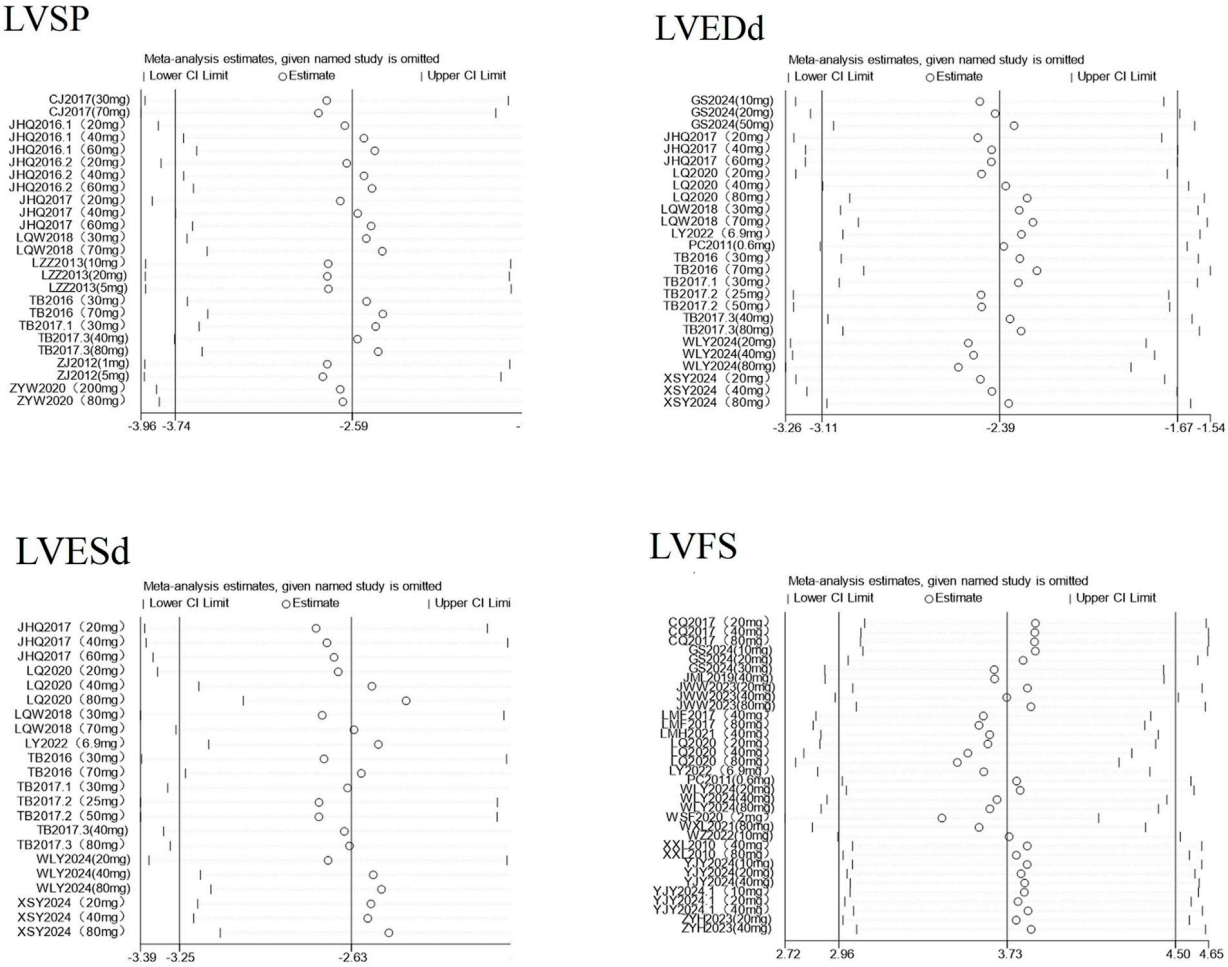
Leave-one-out sensitivity analysis plots of partial cardiac indicators.

To ensure the credibility of the conclusions drawn from this meta-analysis, we conducted a sensitivity analysis to assess whether any individual study would significantly affect the overall results. Using RevMan 5.1 software, for indicators with an I^2^ ≥ 50% and more than three included studies, we sequentially excluded each study one by one and separately measured the changes in the pooled effect size and heterogeneity after each exclusion see Figure 6. The analysis results showed that regardless of which study was removed, there was no significant fluctuation in the overall pooled effect size or heterogeneity, indicating that the results of this meta-analysis have good stability. The corresponding plot is shown in Supplementary Material 2.

**FIGURE 7:**
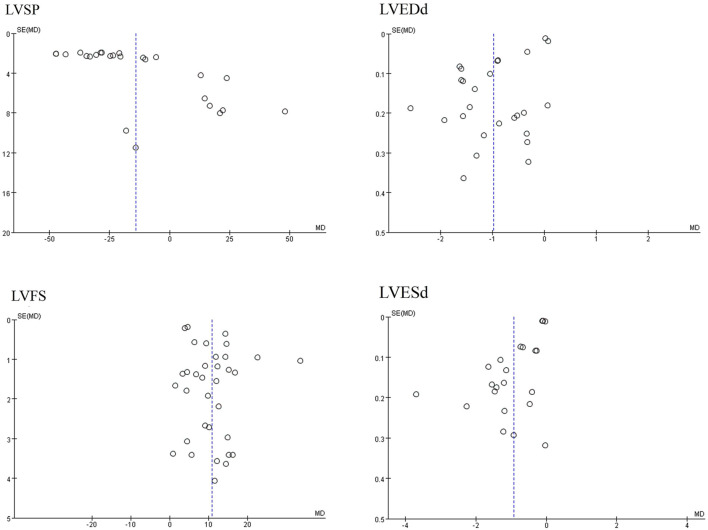
Funnel plots of partial cardiac indicators.

**FIGURE 8:**
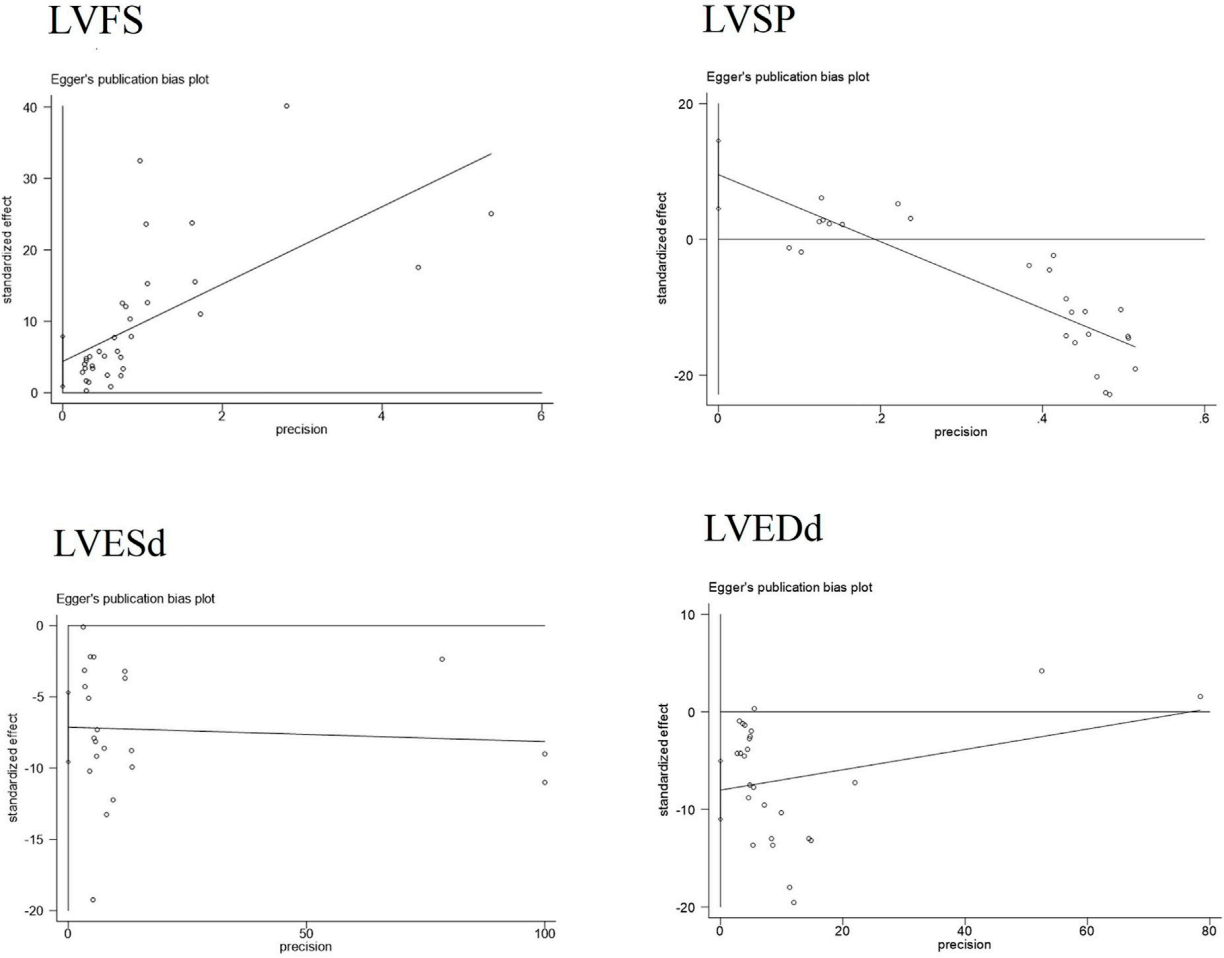
Egger’s plots of partial cardiac indicators.

Publication bias serves as a method to examine potential biases in the outcomes of systematic reviews. In this study, we comprehensively employed funnel plots, Begg’s test, and Egger’s test to evaluate the overall extent of publication bias among the included studies. Specifically, the funnel plots for four indicators—LVEDd, LVESd, LVFS, and LVSP—exhibited obvious asymmetry see Figure 7. Further application of Begg’s and Egger’s tests to these four indicators yielded *P*-values all below 0.05, suggesting the presence of significant publication bias see Figure 8. The corresponding plot is shown in Supplementary Material 2.

**FIGURE 9:**
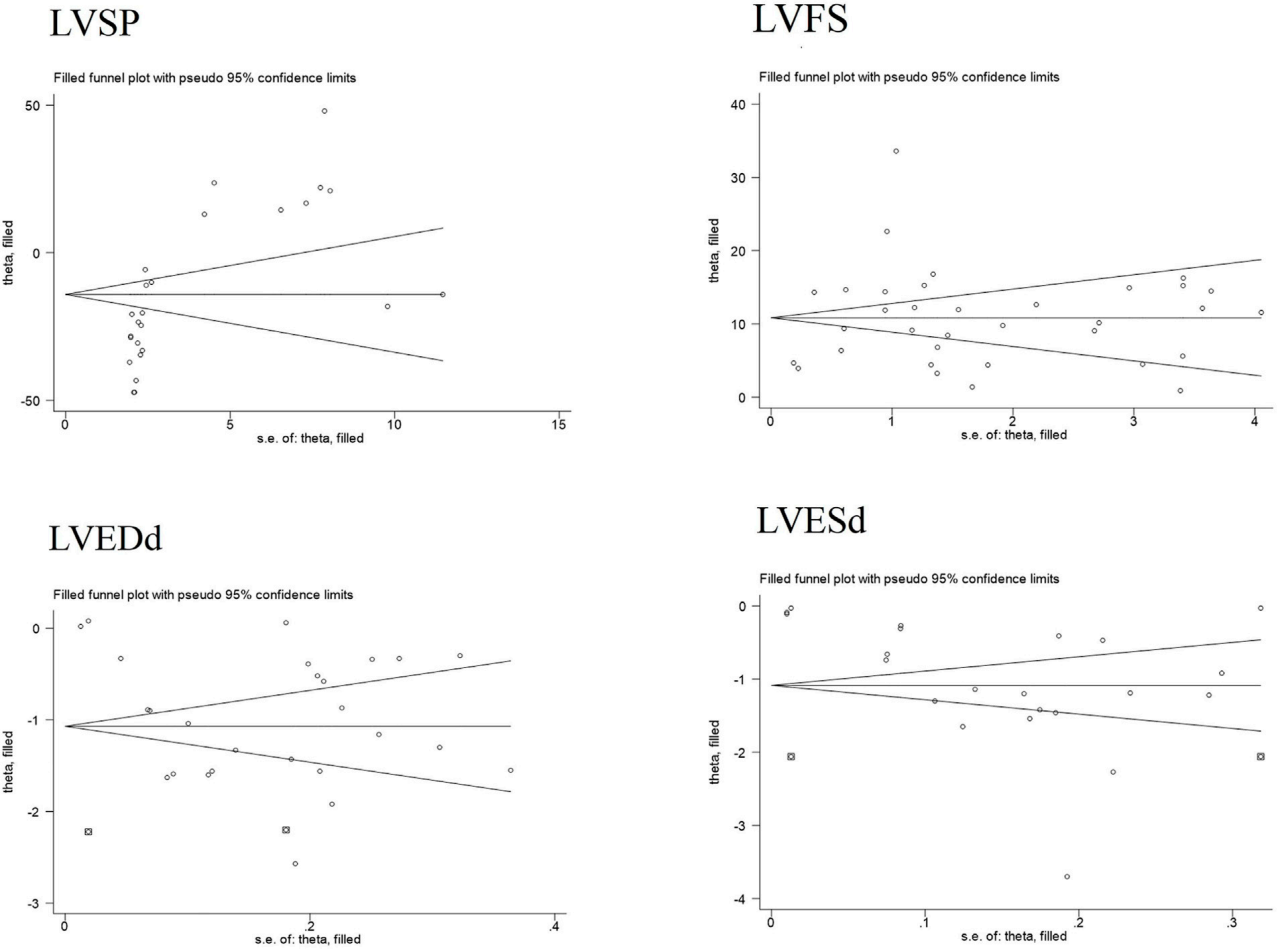
Trim-and-fill analysis plots of cardiac indicators.

Additionally, for the LVESd and LVEDd indices, we incorporated two pieces of virtual data (marked as squares in the figure) using the trim-and-fill method to assess the impact of missing studies on the pooled results. The findings indicated that no reversal occurred, leading to the comprehensive conclusion that the results of these indices exhibit good robustness. For the LVFS and LVSP indices, the trim-and-fill method did not detect any missing studies caused by publication bias see Figure 9. Based on this, we further explored the sources of heterogeneity through meta-regression and subgroup analysis to more thoroughly unravel the potential influencing factors of the effect size.

### 3.3 Meta-regression

In the present study, meta-regression analyses were conducted on indicators exhibiting high statistical heterogeneity with 10 or more included studies. Using species type, administration dose, and administration duration as covariates, these analyses aimed to explore potential sources of heterogeneity. The results demonstrated that species-related factors exerted a significant regulatory effect on LVPWd and CVF (*P* < 0.05); administration dose significantly influenced the effect sizes of LVEDd, LVEDp, LVPWd, and CVF (*P* < 0.05); and administration duration, in turn, exerted a significant regulatory influence on the effect sizes of LVFS, LVEDp, LVPWd, and +dp/dtmax (*P* < 0.05). Species, dose, and duration, operating through distinct mechanisms, emerged as significant contributors to the heterogeneity observed in the aforementioned indicators. Meanwhile, indicators such as IVSd, LVIDs, and body weight, due to having fewer than 10 included studies, lacked adequate data support. The sources of their heterogeneity require subsequent analysis with an expanded sample size.

### 3.4 Subgroup analysis

Additionally, given the significant heterogeneity in studies examining astragaloside IV’s effects on outcome indicators, we conducted concurrent subgroup analyses based on the basic characteristics of the included literature. These analyses aimed to further explore, validate, and refine the conclusions drawn from the meta-regression.

#### 3.4.1 Species subgroup analysis of astragaloside IV in rodent MF models

Subgroup analyses were performed based on experimental animal types, restricted to subgroups with ≥3 included studies. For indicators such as CVF, LVEDd, LVPWd, HR, and TNF-α, the number of mouse studies was small (1–2 articles); after exclusion, AS-IV still significantly reduced these indicators in rat models, consistent with pre-exclusion results. For LVIDd, after excluding studies on rats (only two articles), no significant intervention effect was observed, which remained unchanged. Studies on LVEF and LVFS included data from both rats and mice, and subgroup analyses for each species showed significant improvements. The core effects remained stable after excluding literature on different species, indicating that the effect of AS-IV intervention is consistent across species. This provides evidence for clinical translation. The corresponding plot is shown in Supplementary Material 3.

#### 3.4.2 Dose subgroup analysis of astragaloside IV in rodent MF models

Subgroup analysis based on differences in AS-IV administration doses (with ≥3 included studies per subgroup) revealed a dose-dependent response of cardiac structural parameters to astragaloside IV: reduction in CVF at 20–60 mg/kg, with effects strengthening as the dose increased; improvements in LVESd and LVEDd at 30–80 mg/kg (higher doses yielding superior outcomes); reduction in LVPWd at 30–40 mg/kg and in LVMI at 40–80 mg/kg, with both effects intensifying with increasing doses. For hemodynamic parameters, 10–80 mg/kg elevated LVFS and LVEF (more pronounced effects at higher doses); 10–70 mg/kg reduced LVEDp in a dose-dependent manner; 30–60 mg/kg lowered LVSP; and 10–60 mg/kg increased ±dp/dtmax. Regarding physiological and biochemical parameters, all tested doses reduced LDH levels and TNF-α expression, while HR showed an overall decreasing trend. In summary, astragaloside IV showed predominantly positive regulatory effects on the parameters examined, with clear dose dependence observed for indicators including CVF, LVFS, and LVEF, supporting the exploration of optimal dosing strategies. The corresponding plot is shown in Supplementary Material 3.

#### 3.4.3 Duration subgroup analysis of astragaloside IV in rodent myocardial fibrosis models

Subgroup analysis by administration duration (with ≥3 included studies per subgroup) showed that 28-day and 56-day interventions reduced CVF, with the 56-day intervention yielding superior effects, indicating a time-effect relationship. A 56-day intervention improved indicators such as LVEDd, LVESd, LVMI, and LVPWd with significant efficacy, while a 28-day intervention had a limited impact on LVIDd. Among hemodynamic parameters, 14-day, 28-day, and 56-day interventions increased ±dp/dtmax, LVEF, and LVFS, and decreased LVEDp and LVSP; the 56-day intervention showed more significant effects, suggesting a correlation between duration and functional improvement. A 28-day intervention significantly reduced LDH, confirming its role in improving myocardial injury and providing support for related research. The corresponding plot is shown in Supplementary Material 3.

## 4 Discussion

This meta-analysis shows that AS-IV can effectively reduce CVF, LVESd, LVEDd, IVSd, LVPWd, and LVMI. It also significantly lowers LVEDp and LVSP, while boosting LVFS, LVEF, and ±dp/dtmax. In addition, AS-IV markedly decreases blood levels of LDH and TNF-α, regulates HR in rodent models, and promotes weight gain. Notably, it has no significant impact on LVIDd and LVIDs. Previous basic research on astragaloside IV’s anti-myocardial fibrosis effects has been scattered and lacked comprehensive integration (Li et al., 2023b; Shan et al., 2024).

**FIGURE 10:**
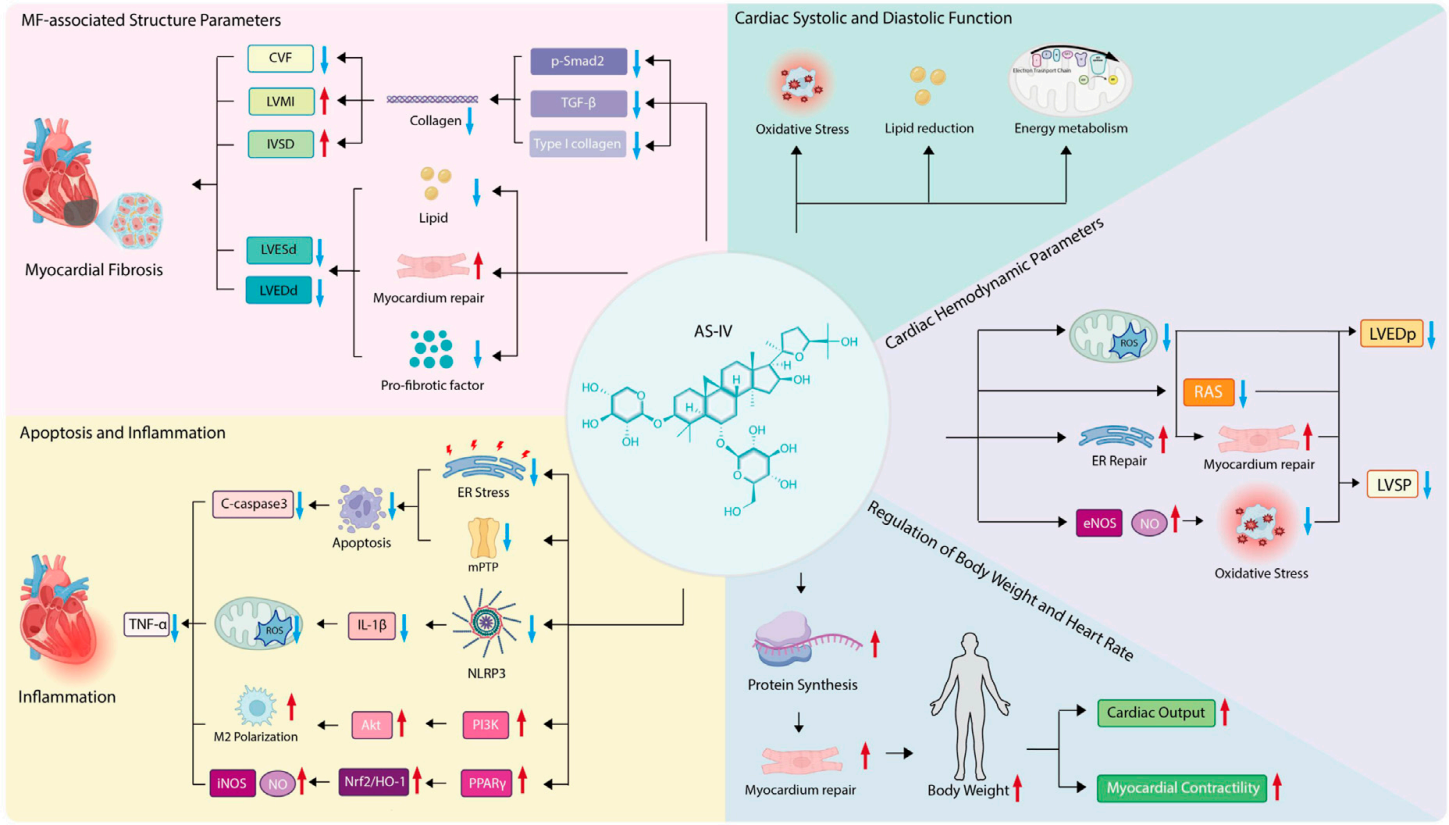
Schematic diagram of the mechanisms and pathways of AS-IV acting on myocardial fibrosis.

This study systematically synthesizes existing evidence, offering a theoretical basis and strategic guidance for the clinical use of AS-IV in cardiac diseases. It also helps assess the compound’s safety and therapeutic efficacy, laying the groundwork for future human clinical trials and drug development efforts. To further clarify the complex regulatory network through which AS-IV exerts its anti-fibrotic effects, a schematic diagram is provided below (Figure 10), which visually summarizes its multi-targeted mechanisms and key regulatory pathways in mitigating myocardial fibrosis.

### 4.1 Effects of AS-IV on structural parameters related to MF

CVF stands as a key histological metric for gauging MF severity, offering a quantitative readout of fibrotic burden by assessing collagen content in myocardial tissue. Higher CVF values signify greater collagen accumulation and more marked fibrotic features (Lang et al., 2025). The current study showed that AS-IV notably lowers CVF, pointing to its potential to alleviate MF. Moreover, these findings suggest CVF regulation in MF ties to the TGF-β/Smad signaling pathway (Xu et al., 2023).

In rat models with MF, CVF was significantly elevated alongside upregulated expression of TGF-β1, phosphorylated Smad2 (P-Smad2), and type I collagen—changes that in turn promote collagen synthesis and deposition (Zeng et al., 2023). These results mirror those from hypoxia-induced myocardial hypertrophy models, where AS-IV reduces collagen deposition (particularly type I collagen) by inhibiting the TGF-β1/Smad2 pathway, lowers CVF, and also increases IVSd and LVMI (Zhang et al., 2024).

Meanwhile, increases in LVESd and LVEDd raise myocardial wall tension, in turn exacerbating myocardial injury and fibrosis. Reducing LVESd and LVEDd thus plays a key role in improving ventricular perfusion. AS-IV has been shown to mitigate ventricular dilation by repairing myocardial damage, boosting contractile function, reducing lipid accumulation, and downregulating pro-fibrotic factor expression (Li et al., 2023a). Additionally, its derivative HHQ16 acts directly on cardiomyocytes, helping reverse myocardial hypertrophy and post-infarction ventricular remodeling, reducing LVESd and LVEDd, and significantly improving cardiac function (Wan et al., 2023).

Taken together, AS-IV regulates CVF, lowers such markers as LVESd, LVEDd, IVSd, and LVMI, and inhibits activation of the TGF-β/Smad pathway—effects that in turn reduce collagen buildup and effectively curb MF.

### 4.2 Regulatory effects of AS-IV on myocardial cell apoptosis and inflammatory response

TNF-α is a core pro-inflammatory mediator, and AS-IV can improve its level. It may alleviate 2-deoxy-D-glucose (2-DG)-induced apoptosis in PC12 cells by inhibiting endoplasmic reticulum stress and blocking the opening of the mitochondrial permeability transition pore (mPTP), thereby significantly reducing the expression of the apoptotic marker Caspase-3 (Wan et al., 2023) and further mitigating the inflammatory response. Furthermore, it can promote M2 microglial polarization by activating the PI3K/AKT signaling pathway, thereby alleviating neuroinflammation and cerebral damage (Wang Z. et al., 2024). These findings suggest that AS-IV may exert cardioprotective effects through similar molecular mechanisms.

In a lipopolysaccharide (LPS)-induced cardiac dysfunction model, AS-IV dampens NLRP3 inflammasome activation, reducing the release of pro-inflammatory cytokines like IL-1β. At the same time, it reins in mitochondrial function and curbs the production of reactive oxygen species (ROS) (Fu et al., 2020; Feng et al., 2021). It also increases the PPARγ signaling pathway, strengthens the Nrf2/HO-1 antioxidant pathway, and reduces excessive iNOS and nitric oxide production (Liang et al., 2023).

These mechanisms point to AS-IV’s ability to exert a synergistic regulatory effect on cardiomyocyte apoptosis and inflammatory responses via multi-target actions. Its impacts span antioxidation, mitochondrial protection, and metabolic reprogramming—all of which lay a theoretical groundwork for its use in cardiovascular diseases (Chen et al., 2021; Zaman et al., 2022; Zhai et al., 2024).

### 4.3 Ameliorative effects of AS-IV on cardiac hemodynamic parameters

AS-IV reduces LVEDp significantly by boosting myocardial diastolic function. In myocardial ischemia-reperfusion (I/R) injury models, pre-treating with AS-IV reduces microvascular leakage (MVL), which in turn eases cardiac edema and diastolic dysfunction, consistent with the drop in LVEDp (He et al., 2022). At the same time, AS-IV can repair mitochondrial function, curb ROS buildup, and improve calcium handling in cardiomyocytes, all of which lower LVEDp (Wang et al., 2024b; Wang et al., 2024c).

In sepsis-induced cardiomyopathy models, AS-IV increases myocardial contractility by restoring mitochondrial balance and endoplasmic reticulum function (Su et al., 2022). It also increases endothelial nitric oxide synthase (eNOS) activity, spurring more nitric oxide (NO) production. This mitigates oxidative stress-related myocardial damage and keeps LVSP stable (Meng et al., 2021; Leng et al., 2020).

Studies show AS-IV improves ventricular geometric remodeling by shrinking LVEDp and LVSP. In chronic kidney disease (CKD) models with concurrent myocardial injury, AS-IV eases ventricular dilation by blocking the renin-angiotensin system (RAS) and fibrotic signaling pathways (Li et al., 2023b; Li et al., 2022). Notably, combining AS-IV with other active compounds like tanshinone IIA synergistically enhances LVEDp and LVSP, yielding a more robust cardioprotective effect (Zhai et al., 2024).

In short, AS-IV eases cardiac dysfunction by comprehensively regulating LVEDp and LVSP through a multi-target mechanism.

### 4.4 Enhancing effects of AS-IV on cardiac systolic and diastolic function

AS-IV, the primary bioactive component in *Astragalus*, delivers a range of cardioprotective effects by boosting both systolic and diastolic heart function. Under hypoxic conditions, it significantly eases myocardial hypertrophy and cardiac injury—effects likely tied to curbing oxidative stress and regulating energy metabolism (Zhang et al., 2024).

What is more, in diabetic cardiomyopathy (DCM), AS-IV helps repair myocardial damage, increases contractile function, and reduces lipid buildup (Li et al., 2023a). It also enhances vascular relaxation by reversing oxidative stress-induced endothelial dysfunction, doing so by increasing eNOS activity and boosting NO levels.

AS-IV markedly improves cardiac systolic and diastolic function through multi-target, multi-pathway actions. Its key mechanisms include antioxidant and anti-inflammatory activity, regulating mitochondrial dynamics, and tweaking various signaling pathways (He et al., 2022).

### 4.5 Potential role of BW and HR regulation in AS-IV-mediated anti-fibrosis

Myocardial cells in underweight individuals often suffer from long-term malnutrition, which impairs their energy metabolism and weakens their self-repair capacity (Fernandez-Patron et al., 2024). Studies show AS-IV can boost body weight in rodent models, hinting that the compound might aid myocardial repair and regeneration by ensuring adequate protein supply, thus slowing fibrosis progression.

Weight gain correlates with preserving key cardiac function markers like myocardial contractility and cardiac output, which may ease fibrosis’s harmful impact on heart performance. What is more, a reduced heart rate can improve myocardial oxygen use, reduce cardiac afterload, and, in turn, slow the progression of MF.

### 4.6 Analysis of limitations

This systematic review has certain limitations: 1) Currently, the pharmacokinetic characteristics of astragaloside IV in humans remain unclear, and the efficacy observed in animal experiments cannot be directly extrapolated to clinical dosing regimens in humans; 2) most animal experiments involve short-term interventions, which do not align with the actual clinical scenario of long-term fibrosis requiring continuous treatment. This may lead to an underestimation of long-term drug efficacy or overlook delayed adverse reactions, thereby affecting the benefit-risk assessment; 3) the pathogenic mechanisms of animal models differ significantly from those of human chronic fibrosis, resulting in model-dependent bias; 4) there is uncertainty regarding the application of allocation concealment in all included studies, and it remains unclear whether implementers of interventions used blinding methods, which introduces considerable selection bias into the results; 5) variations in administration dosages and intervention durations are likely to induce heterogeneity in outcomes, undermining the stability, reliability, and generalizability of the conclusions. It is anticipated that more high-quality literature will be included in future studies to obtain more robust evidence-based medical evidence.

## 5 Conclusion

This meta-analysis found that AS-IV significantly alleviates myocardial injury and oxidative stress in rodent MF models, effectively improving ventricular diastolic and systolic function while reducing cardiomyocyte necrosis and apoptosis. Additionally, BW and HR may indirectly regulate fibrosis progression by influencing myocardial oxygen supply-demand balance. Future studies should adopt standardized experimental designs and dose-optimization strategies, with inclusion of higher-quality evidence to enhance the robustness of findings and support clinical translation.

## Data Availability

The original contributions presented in the study are included in the article/Supplementary Material; further inquiries can be directed to the corresponding author.

## Abbreviations

+dp/dtmax, maximum rate of systolic pressure rise; −dp/dtmax:, maximum rate of diastolic pressure fall; AS-IV, astragaloside IV; BW:, body weight; CI, confidence interval; CVF, collagen volume fraction; HR, heart rate; IVSd, interventricular septal thickness at diastole; LDH, lactate dehydrogenase; LVEDd, left ventricular end-diastolic diameter; LVEDp, left ventricular end-diastolic pressure; LVEF, left ventricular ejection fraction; LVESd, left ventricular end-systolic diameter; LVFS, left ventricular fractional shortening; LVIDd, left ventricular internal diameter at diastole; LVIDs, left ventricular internal diameter at systole; LVMI, left ventricular mass index; LVPWd, left ventricular posterior wall diastolic thickness; LVSP, left ventricular systolic pressure; MD, mean difference; MF, myocardial fibrosis; TNF-α, tumor necrosis factor-alpha.
