# Supplementary material for: The interventional effect of astragaloside IV on rodent models of myocardial fibrosis: a systematic review and meta-analysis
Source: Front Pharmacol. 2025 Sep 22;16:1625774. doi: 10.3389/fphar.2025.1625774 (PMC12497706; doi:10.3389/fphar.2025.1625774)
Supplement: Supplementary file 1 [file Supplementaryfile2.doc]

**Supplementary Materials 2: Funnel Plots**


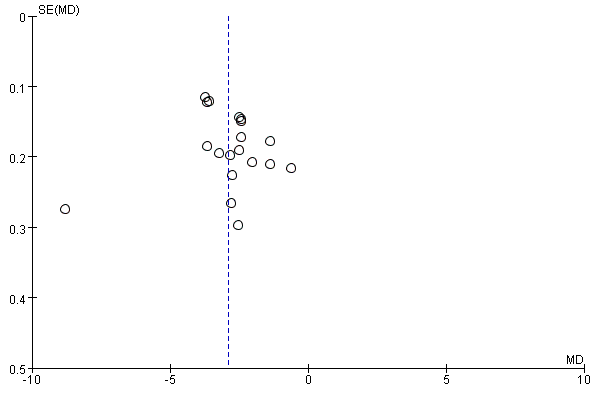


Fig. 1 Funnel plot for evaluating publication bias of the CVF index in the treatment of MF with AS-IV.


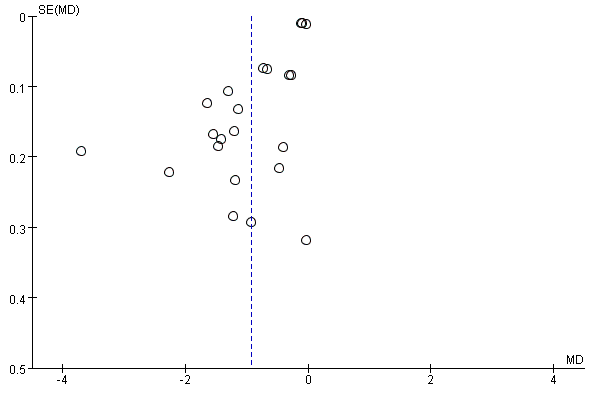


Fig. 2 Funnel plot for evaluating publication bias of the LVESd index in the treatment of MF with AS-IV .


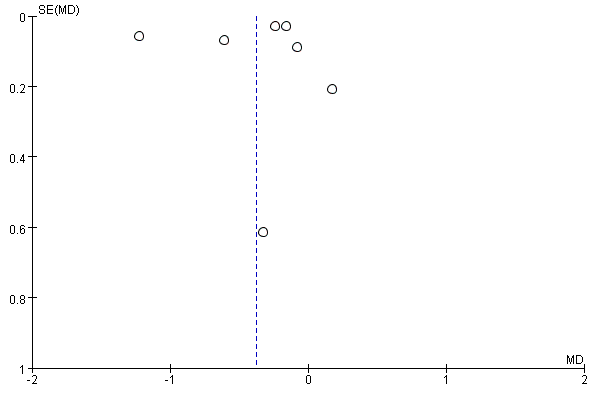


Fig. 3 Funnel plot for evaluating publication bias of the IVSd index in the treatment of MF with AS-IV.


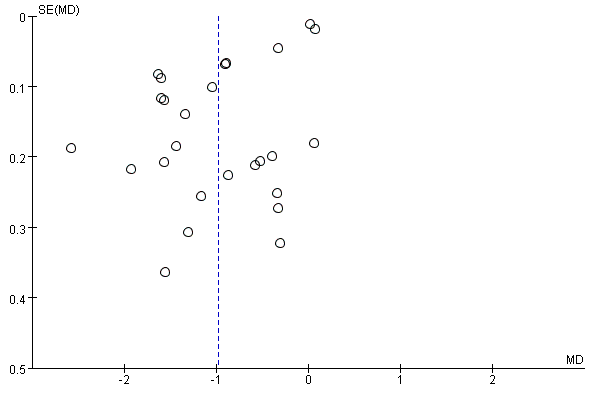


Fig. 4 Funnel plot for evaluating publication bias of the LVEDd index in the treatment of MF with AS-IV


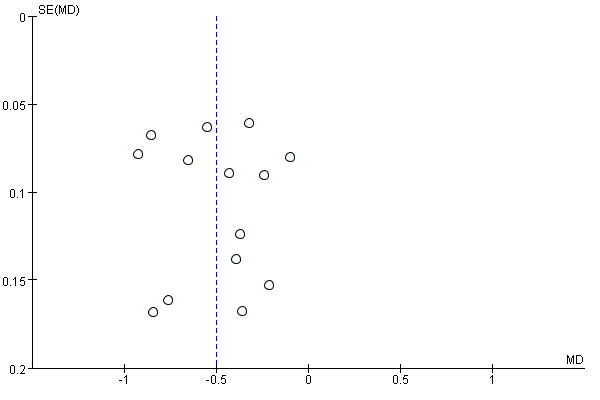


Fig. 5 Funnel plot for evaluating publication bias of the LVMI index in the treatment of MF with AS-IV


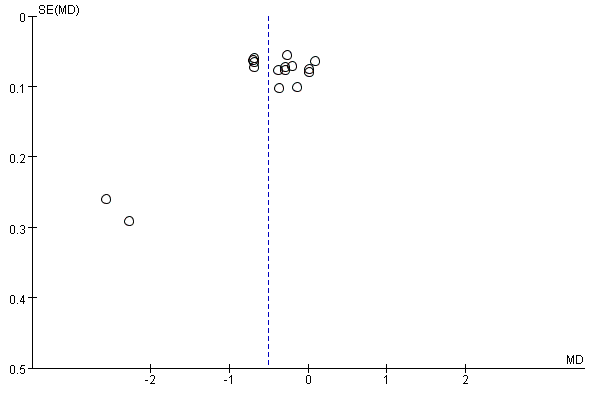


Fig. 6 Funnel plot for evaluating publication bias of the LVPWd index in the treatment of MF with AS-IV


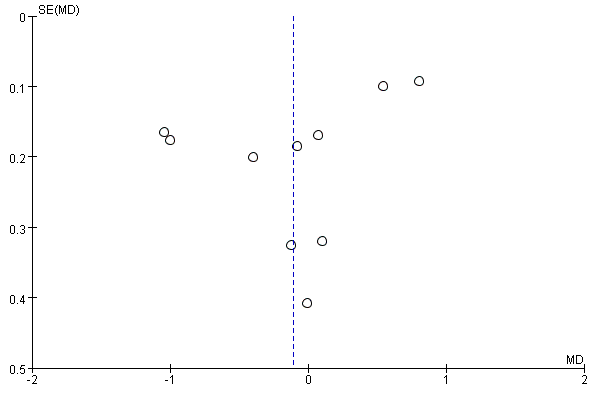


Fig. 7 Funnel plot for evaluating publication bias of the LVIDd index in the treatment of MF with AS-IV


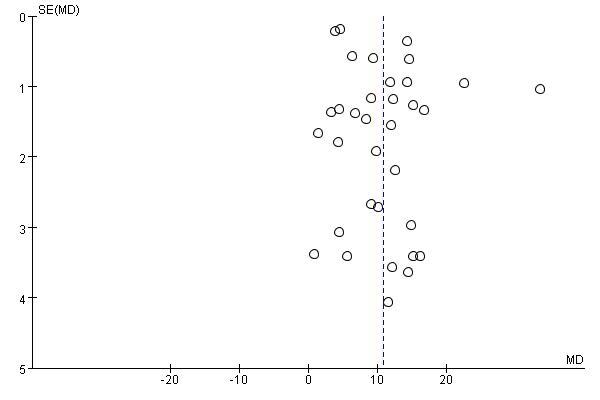


Fig. 8 Funnel plot for evaluating publication bias of the LVFS index in the treatment of MF with AS-IV


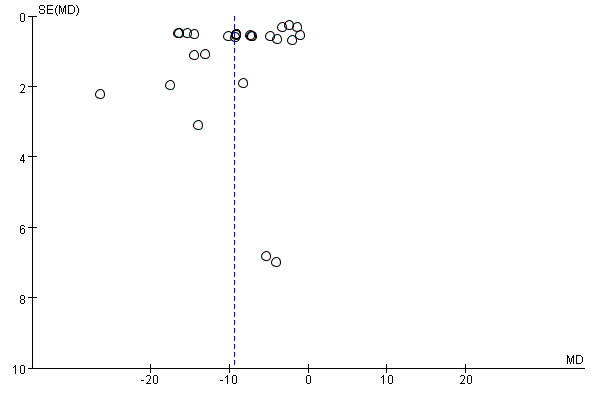


Fig. 9 Funnel plot for evaluating publication bias of the LVEDp index in the treatment of MF with AS-IV


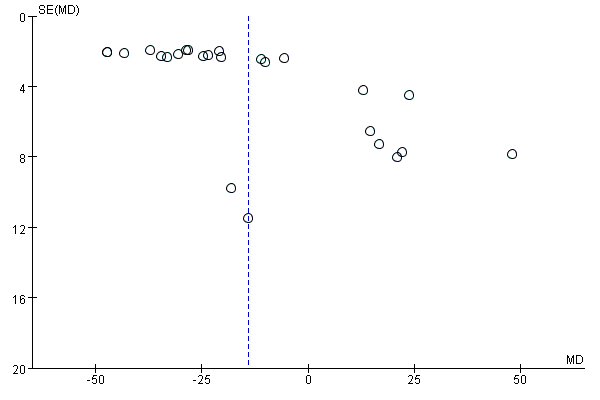


Fig. 10 Funnel plot for evaluating publication bias of the LVSP index in the treatment of MF with AS-IV


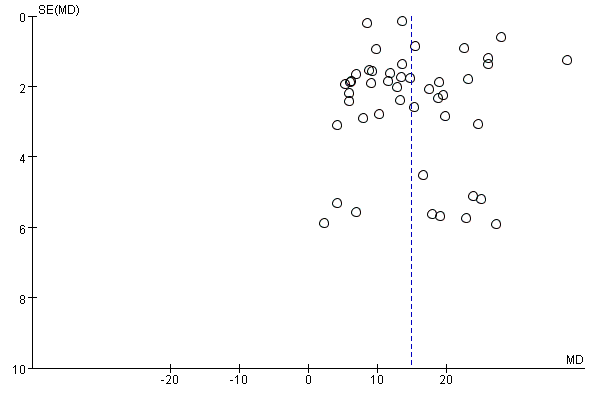


Fig. 11 Funnel plot for evaluating publication bias of the LVEF index in the treatment of MF with AS-IV


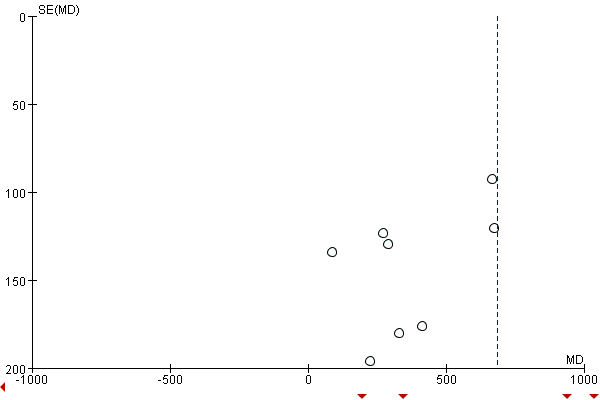


Fig. 12 Funnel plot for evaluating publication bias of the +dp/dtmax index in the treatment of MF with AS-IV


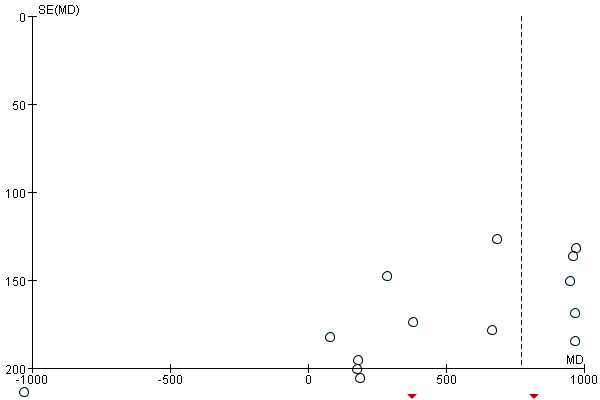


Fig. 13 Funnel plot for evaluating publication bias of the -dp/dtmax index in the treatment of MF with AS-IV


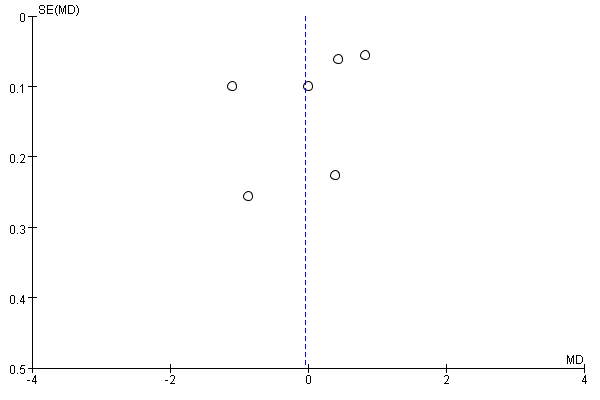


Fig. 14 Funnel plot for evaluating publication bias of the LVIDs index in the treatment of MF with AS-IV


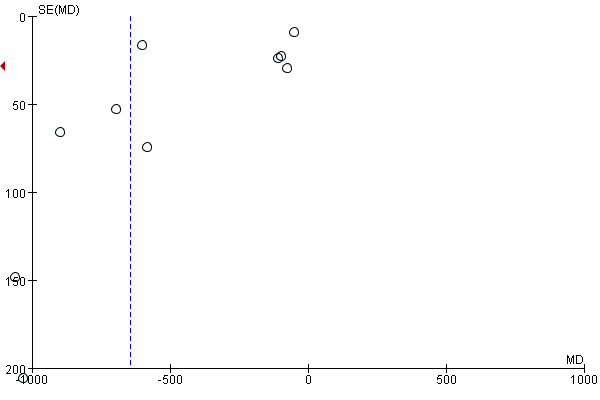


Fig. 15 Funnel plot for evaluating publication bias of the LDH index in the treatment of MF with AS-IV


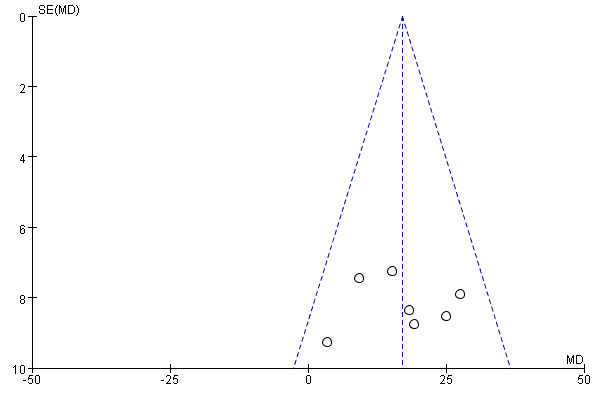


Fig. 16 Funnel plot for evaluating publication bias of the BW index in the treatment of MF with AS-IV


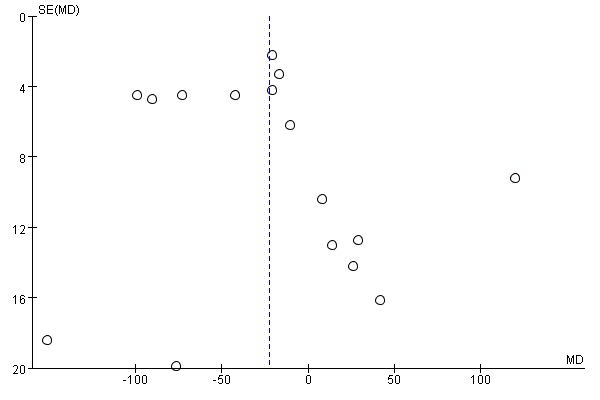


Fig. 17 Funnel plot for evaluating publication bias of the HR index in the treatment of MF with AS-IV


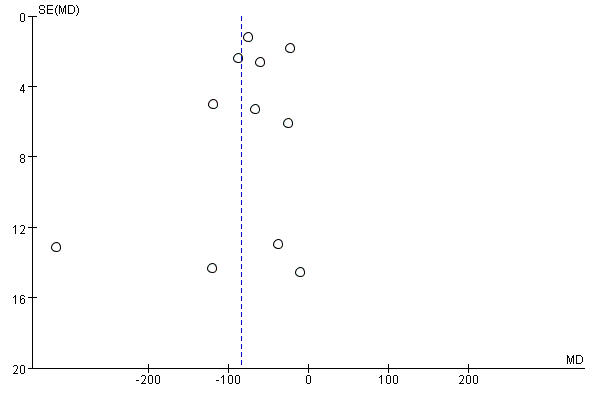


Fig. 18 Funnel plot for evaluating publication bias of the TNF -α index in the treatment of MF with AS-IV

**Supplementary Materials 2: Egger's Plots**

Fig. 19 Egger’s Plots for detecting publication bias of the CVF index in the treatment of MF with AS-IV.

Fig. 20 Egger’s Plots for detecting publication bias of the LVESd index in the treatment of MF with AS-IV.

Fig. 21 Egger’s Plots for detecting publication bias of the LVEDd index in the treatment of MF with AS-IV.

Fig. 22 Egger’s Plots for detecting publication bias of the IVSD index in the treatment of MF with AS-IV.

.

Fig. 23 Egger’s Plots for detecting publication bias of the LVMI index in the treatment of MF with AS-IV.

Fig. 24 Egger’s Plots for detecting publication bias of the LVPWd index in the treatment of MF with AS-IV.

Fig. 25 Egger’s Plots for detecting publication bias of the LVIDd index in the treatment of MF with AS-IV.

Fig. 26 Egger’s Plots for detecting publication bias of the LVFS index in the treatment of MF with AS-IV.

Fig. 27 Egger’s Plots for detecting publication bias of the LVEDp index in the treatment of MF with AS-IV.

Fig. 28 Egger’s Plots for detecting publication bias of the LVSP index in the treatment of MF with AS-IV.

Fig. 29 Egger’s Plots for detecting publication bias of the LVEF index in the treatment of MF with AS-IV.

Fig. 30 Egger’s Plots for detecting publication bias of the +dp/dtmax index in the treatment of MF with AS-IV.

Fig. 31 Egger’s Plots for detecting publication bias of the -dp/dtmax index in the treatment of MF with AS-IV.

Fig. 32 Egger’s Plots for detecting publication bias of the LVIDs index in the treatment of MF with AS-IV.

、

Fig. 33 Egger’s Plots for detecting publication bias of the LDH index in the treatment of MF with AS-IV.

Fig. 34 Egger’s Plots for detecting publication bias of the BW index in the treatment of MF with AS-IV.

Fig. 35 Egger’s Plots for detecting publication bias of the HR index in the treatment of MF with AS-IV.

Fig. 36 Egger’s Plots for detecting publication bias of the TNF -α index in the treatment of MF with AS-IV.

**Supplementary Material 2: Leave-one-out method**

Fig. 37 Leave - one - out Sensitivity Analysis Plot of LVESd

Fig. 38 Leave - one - out Sensitivity Analysis Plot of LVEF

Fig. 39 Leave - one - out Sensitivity Analysis Plot of LVEDd

Fig. 40 Leave - one - out Sensitivity Analysis Plot of LVMI

Fig. 41 Leave - one - out Sensitivity Analysis Plot of LVFS

Fig. 42 Leave - one - out Sensitivity Analysis Plot of LVEDp

Fig. 43 Leave - one - out Sensitivity Analysis Plot of TNF-α

Fig. 44 Leave - one - out Sensitivity Analysis Plot of IVSd

Fig. 45 Leave - one - out Sensitivity Analysis Plot of LVPWd

Fig. 46 Leave - one - out Sensitivity Analysis Plot of CVF

Fig. 47 Leave - one - out Sensitivity Analysis Plot of LVSP

Fig. 48 Leave - one - out Sensitivity Analysis Plot of LDH

Fig. 49 Leave - one - out Sensitivity Analysis Plot of LVIDs

Fig. 50 Leave - one - out Sensitivity Analysis Plot of LVIDd

Fig. 51 Leave - one - out Sensitivity Analysis Plot of +dp/dtmax

Fig. 52 Leave - one - out Sensitivity Analysis Plot of -dp/dtmax

Fig. 53 Leave - one - out Sensitivity Analysis Plot of BW

Fig. 54 Leave - one - out Sensitivity Analysis Plot of HR

**Supplementary Material 2: Trim-and-Fill Method**

Fig. 55 Trim-and-Fill Plot of LVESd

Fig. 56 Trim-and-Fill Plot of LVEDd

Fig. 57 Trim-and-Fill Plot of LVFS

Fig. 58 Trim-and-Fill Plot of LVSP
