# Supplementary material for: The interventional effect of astragaloside IV on rodent models of myocardial fibrosis: a systematic review and meta-analysis
Source: Front Pharmacol. 2025 Sep 22;16:1625774. doi: 10.3389/fphar.2025.1625774 (PMC12497706; doi:10.3389/fphar.2025.1625774)
Supplement: Supplementary file 2 [file Supplementaryfile3.doc]

**Supplementary Materials 3: Subgroup Analyses by Species**


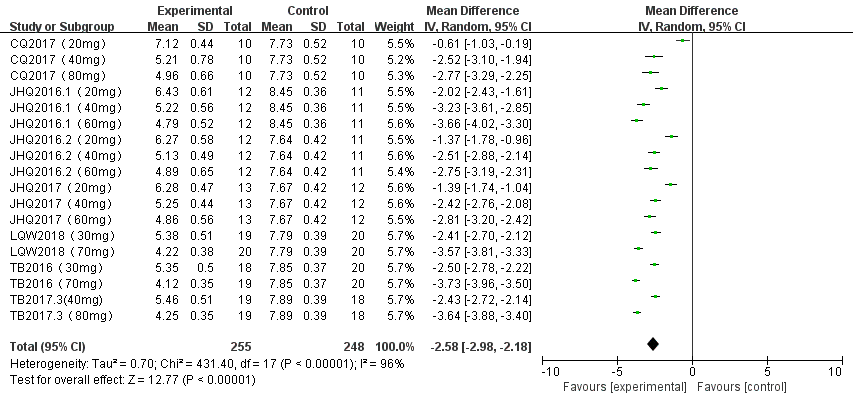


Fig. 1 Species Subgroup Analysis of Astragaloside IV on CVF in Rodent MF Models


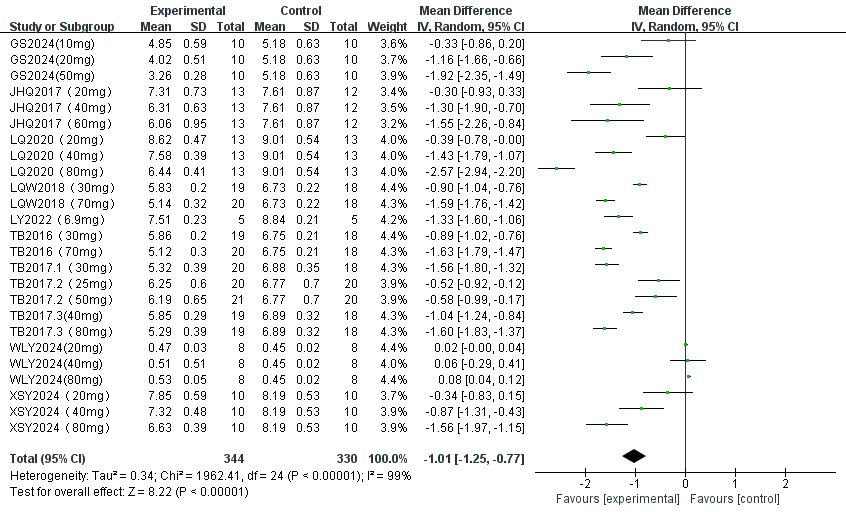


Fig. 2 Species Subgroup Analysis of Astragaloside IV on LVEDd in Rodent MF Models


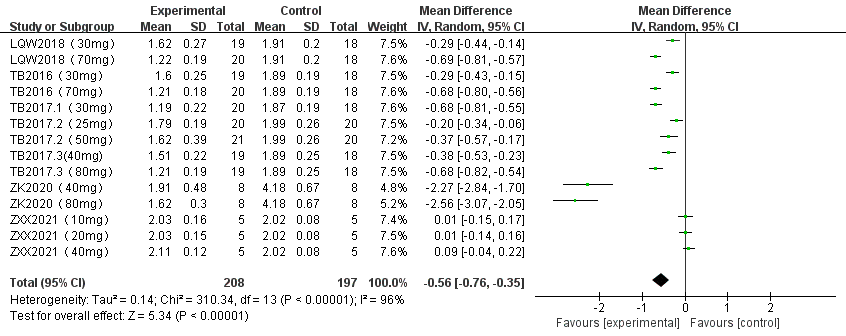


Fig. 3 Species Subgroup Analysis of Astragaloside IV on LVPWd in Rodent MF Models


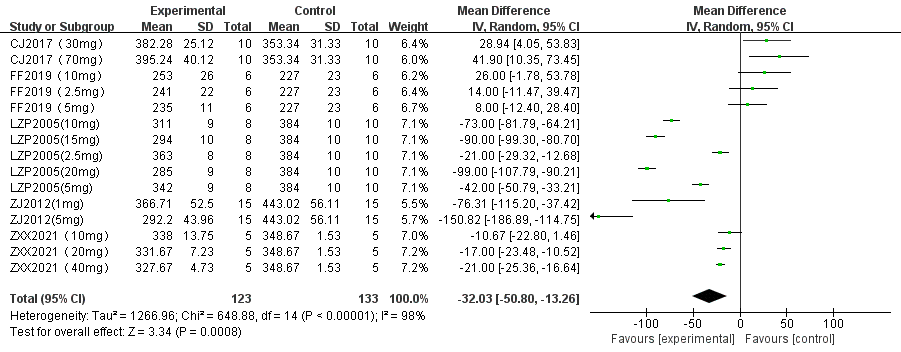


Fig. 4 Species Subgroup Analysis of Astragaloside IV on HR in Rodent MF Models


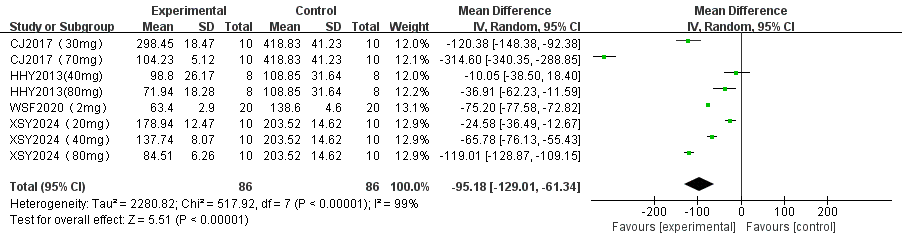


Fig. 5 Species Subgroup Analysis of Astragaloside IV on TNF-α in Rodent MF Models


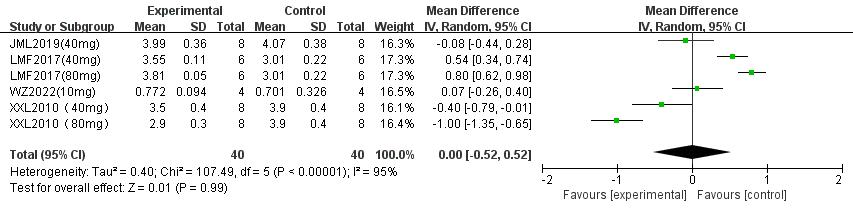


Fig. 6 Species Subgroup Analysis of Astragaloside IV on LVIDd in Rodent MF Models


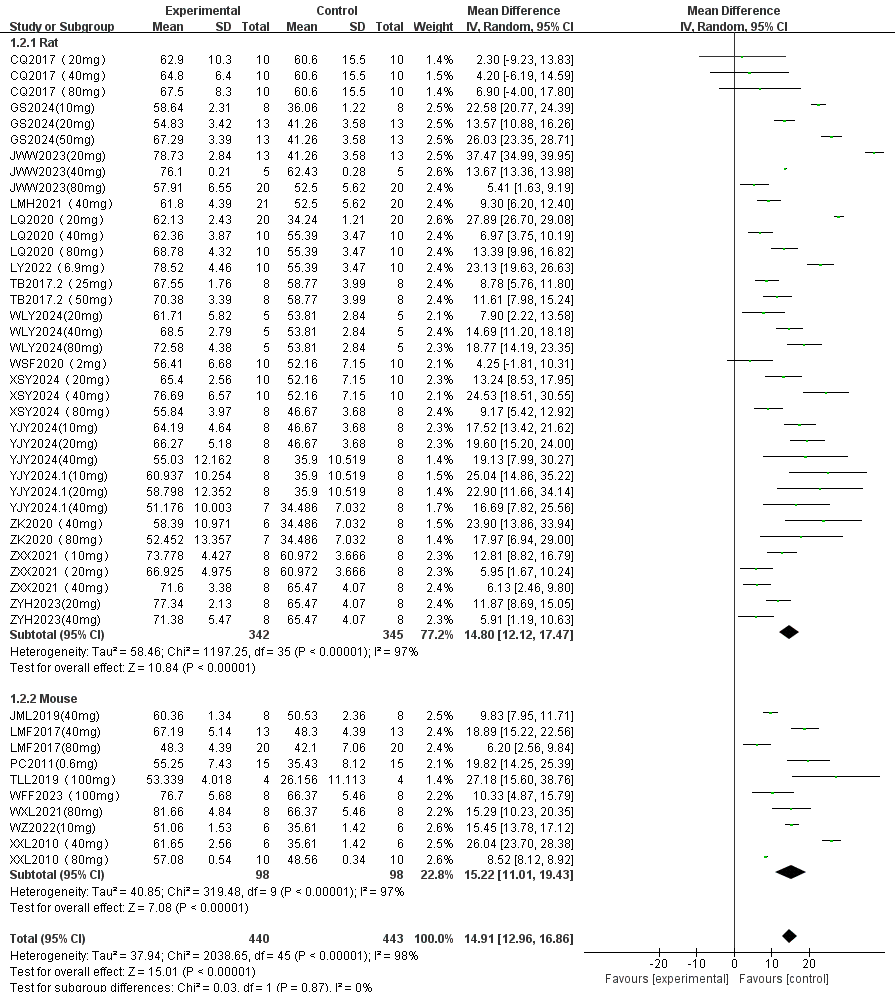


Fig. 7 Species Subgroup Analysis of Astragaloside IV on LVEF in Rodent MF Models


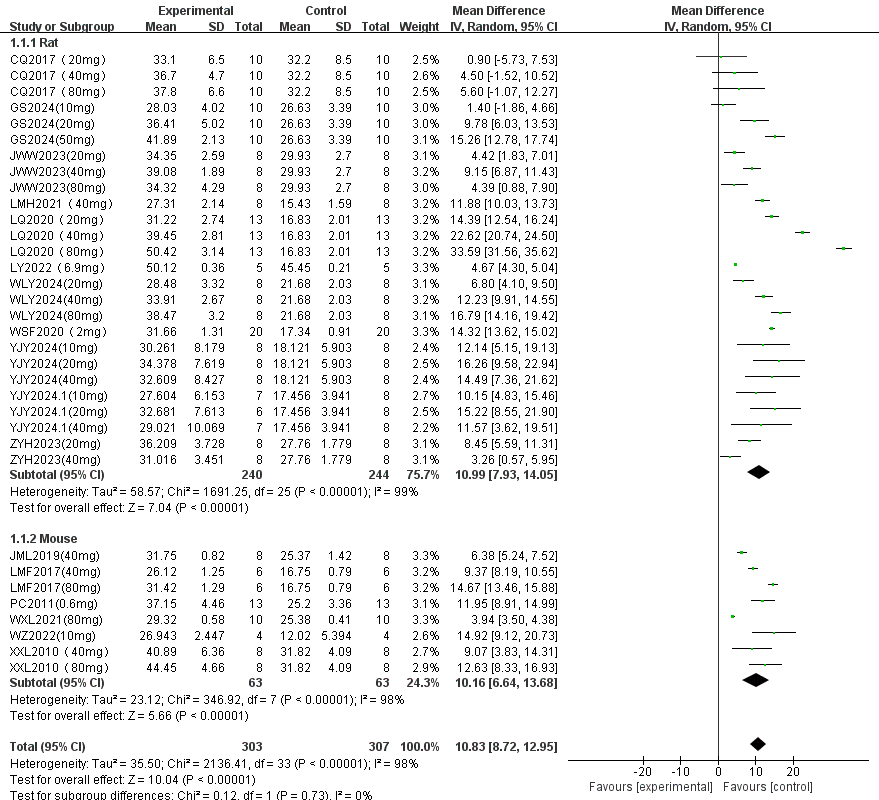


Fig. 8 Species Subgroup Analysis of Astragaloside IV on LVFS in Rodent MF Models

**Supplementary Materials 3: Subgroup Analyses by Dosage**

**
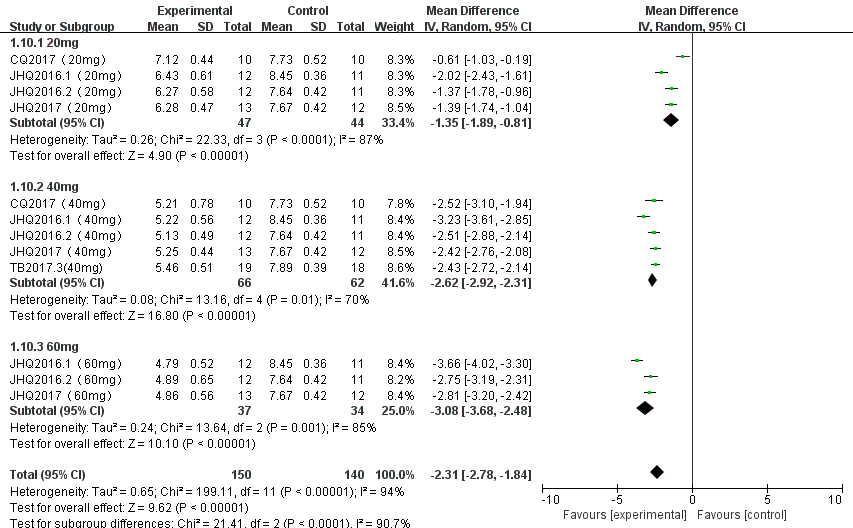
**

Fig. 9 Dosage Subgroup Analysis of Astragaloside IV on CVF in Rodent MF Models


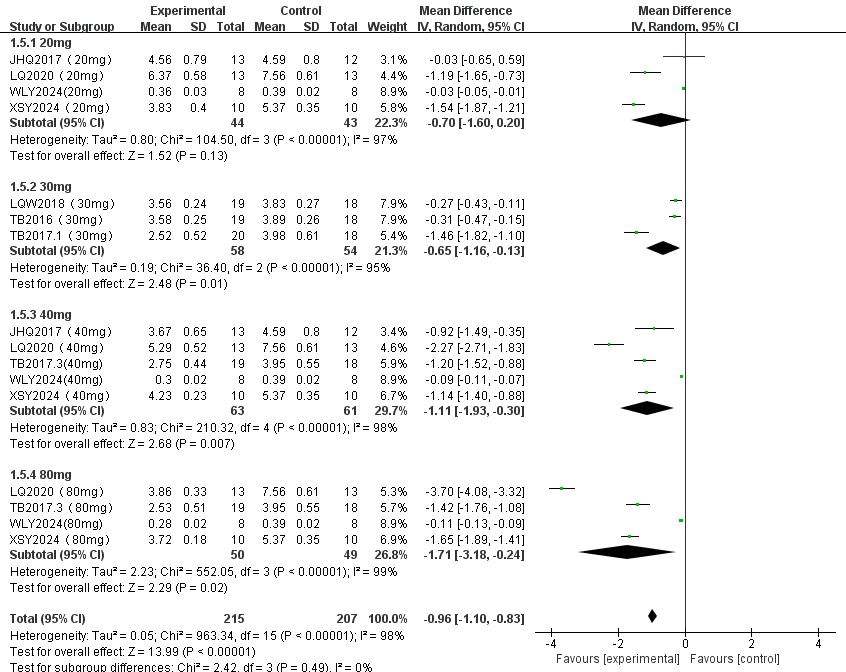


Fig. 10 Dosage Subgroup Analysis of Astragaloside IV on LVESd in Rodent MF Models


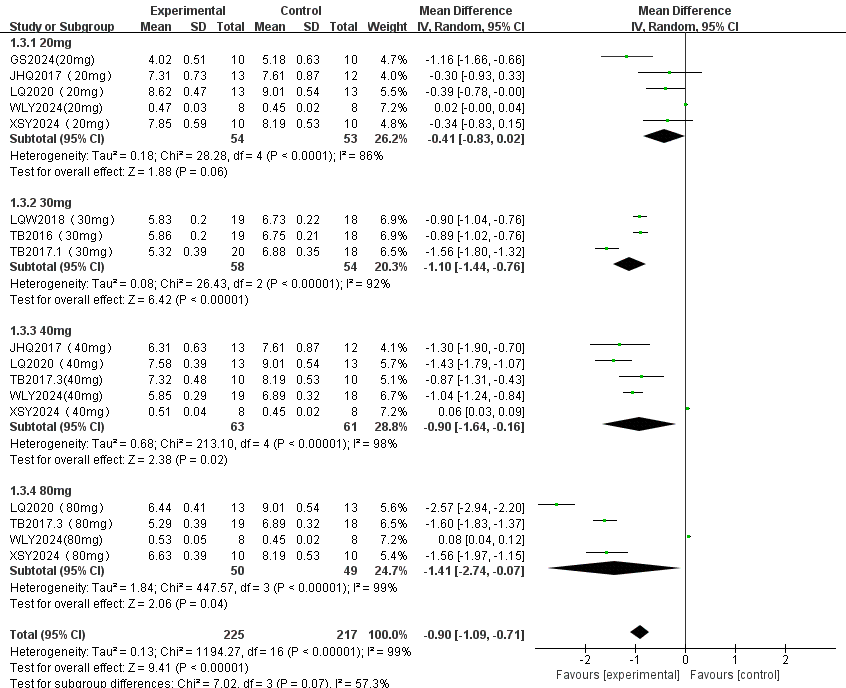


Fig. 11 Dosage Subgroup Analysis of Astragaloside IV on LVEDd in Rodent MF Models


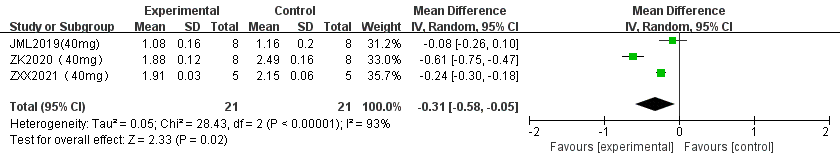


Fig. 12 Dosage Subgroup Analysis of Astragaloside IV on IVSd in Rodent MF Models


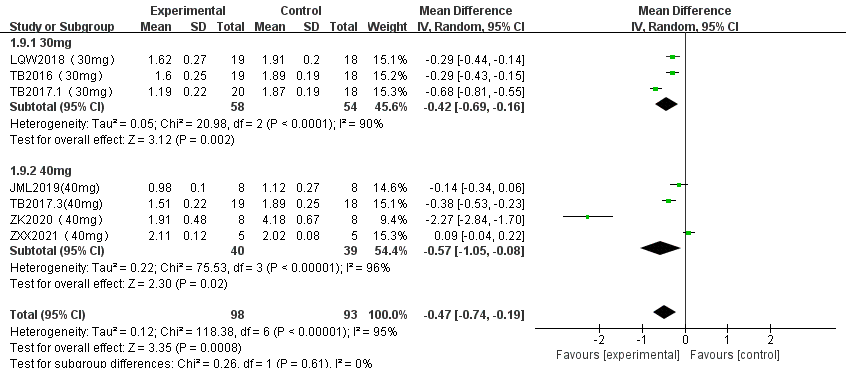


Fig. 13 Dosage Subgroup Analysis of Astragaloside IV on LVPWd in Rodent MF Models


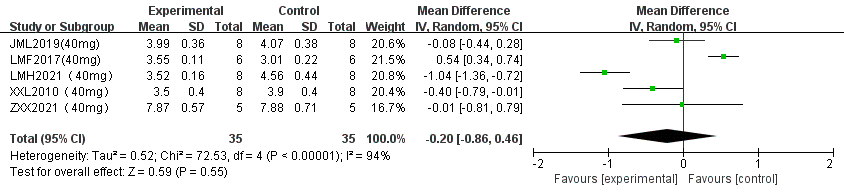


Fig. 14 Dosage Subgroup Analysis of Astragaloside IV on LVIDd in Rodent MF Models


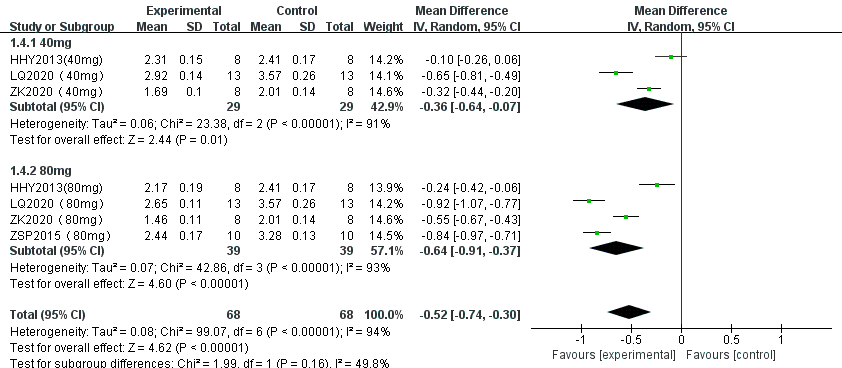


Fig. 15 Dosage Subgroup Analysis of Astragaloside IV on LVMI in Rodent MF Models


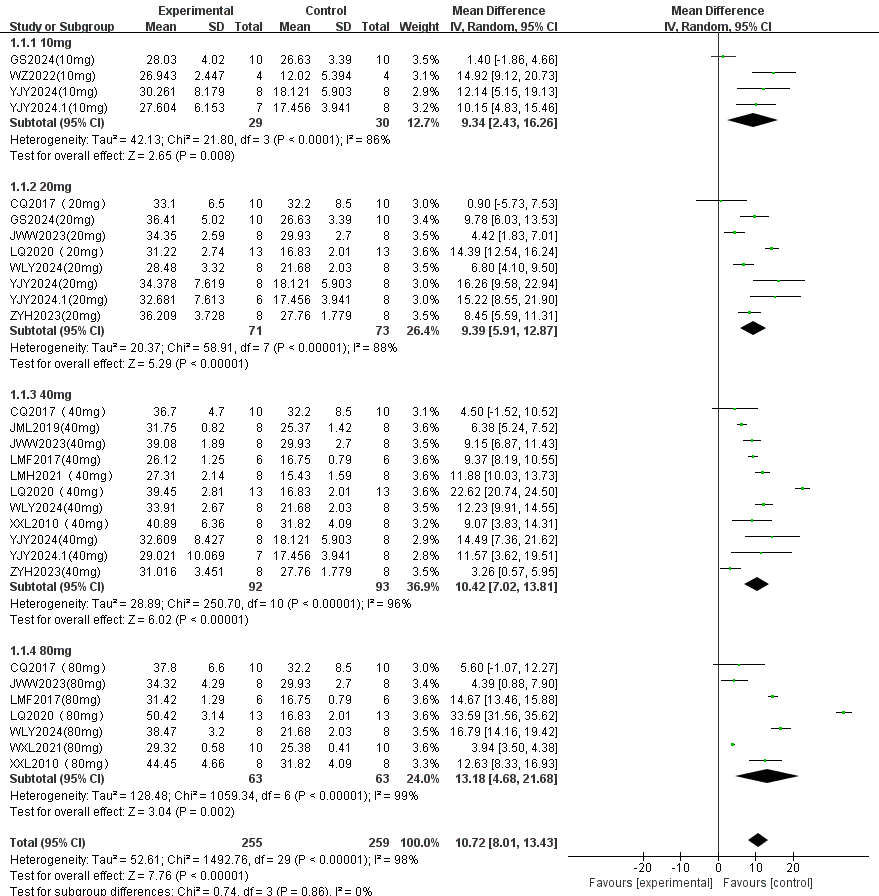


Fig. 16 Dosage Subgroup Analysis of Astragaloside IV on LVFS in Rodent MF Models


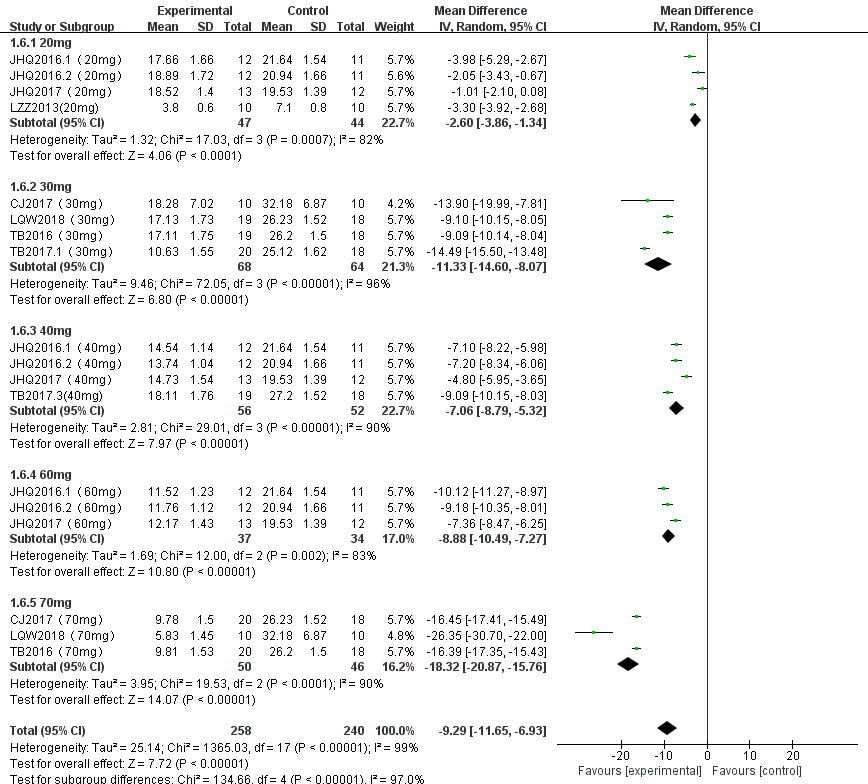


Fig. 17 Dosage Subgroup Analysis of Astragaloside IV on LVEDp in Rodent MF Models


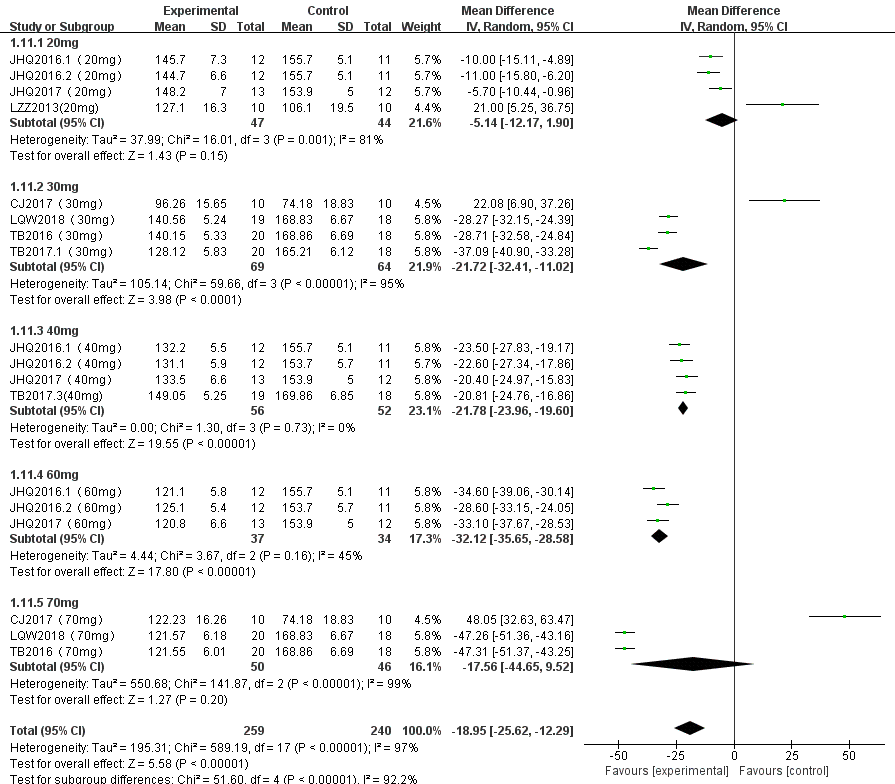


Fig. 18 Dosage Subgroup Analysis of Astragaloside IV on LVSP in Rodent MF Models


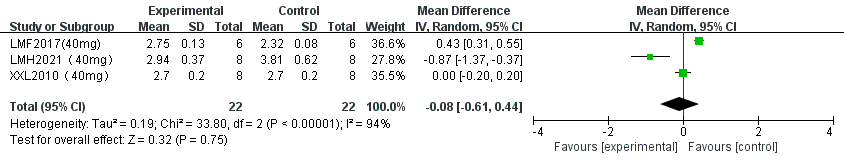


Fig. 19 Dosage Subgroup Analysis of Astragaloside IV on LVIDs in Rodent MF Models


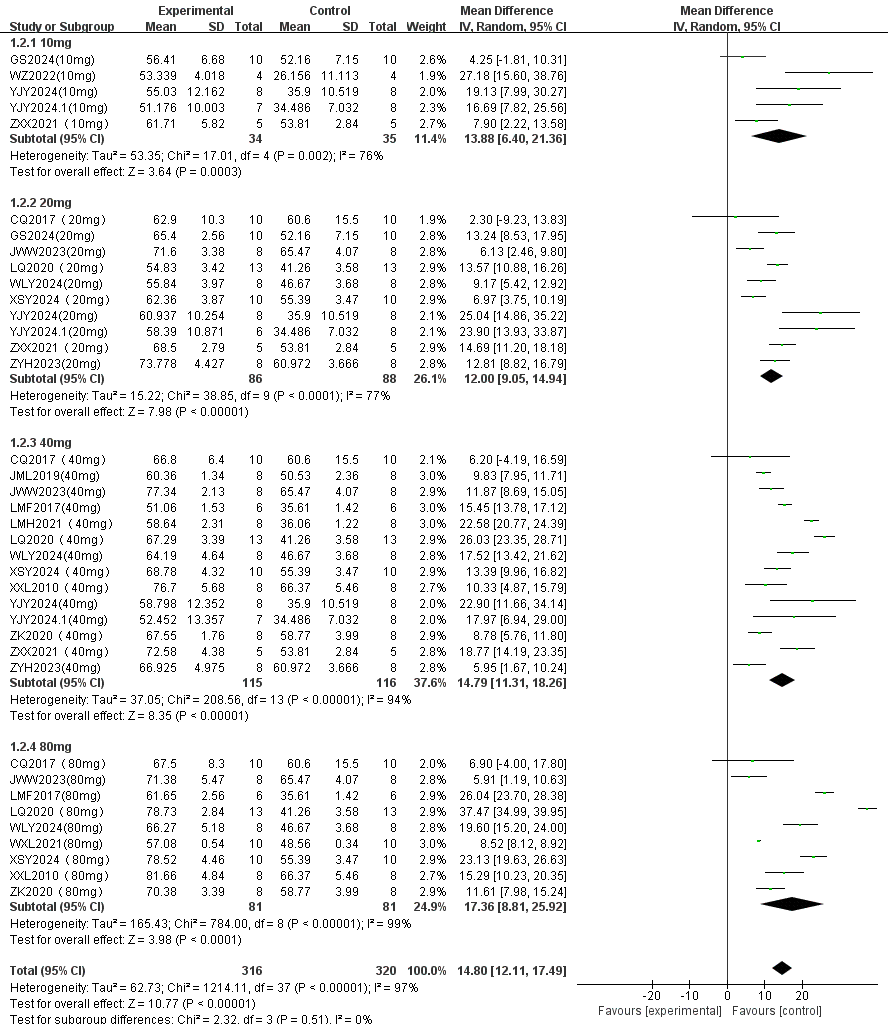


Fig. 20 Dosage Subgroup Analysis of Astragaloside IV on LVEF in Rodent MF Models


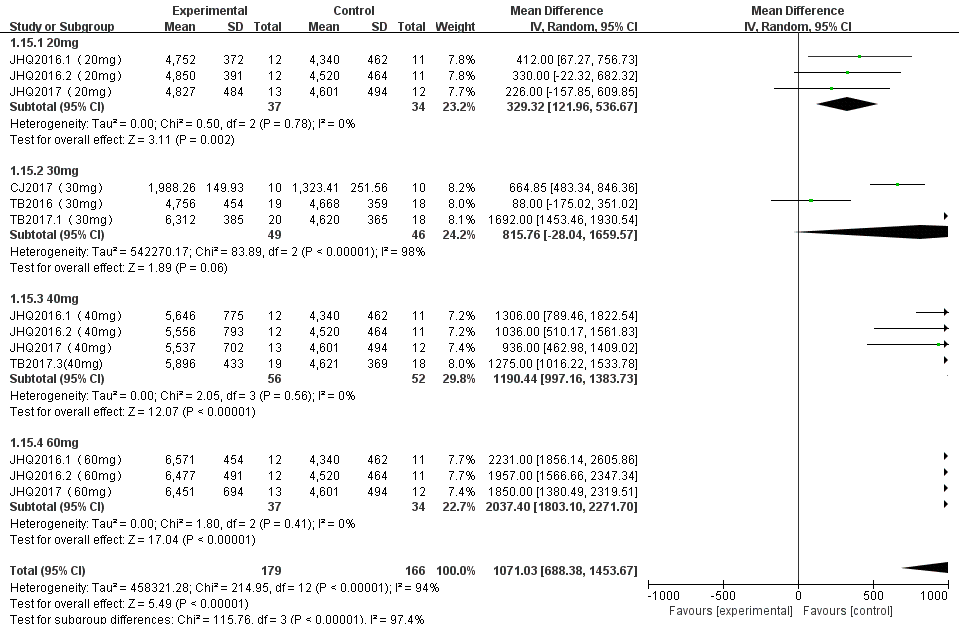


Fig. 21 Dosage Subgroup Analysis of Astragaloside IV on +dp/dtmax in Rodent MF Models


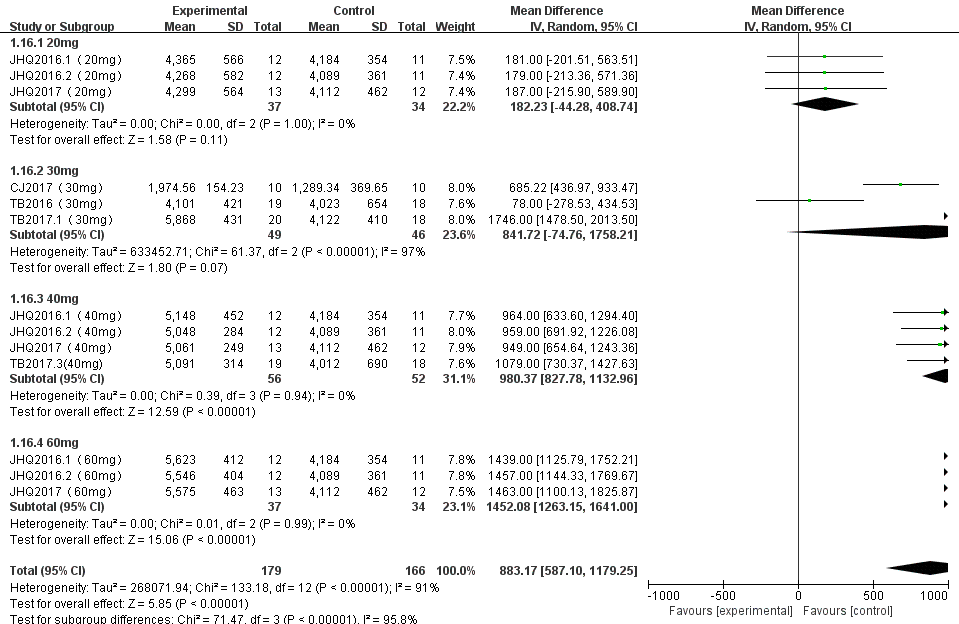


Fig. 22 Dosage Subgroup Analysis of Astragaloside IV on –dp/dtmax in Rodent MF Models


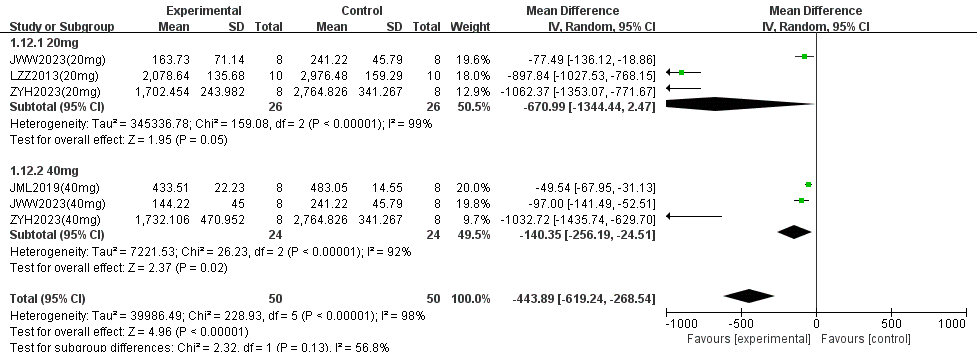


Fig. 23 Dosage Subgroup Analysis of Astragaloside IV on LDH in Rodent MF Models


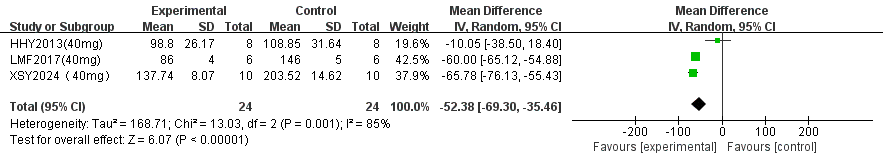


Fig. 24 Dosage Subgroup Analysis of Astragaloside IV on TNF-αin Rodent MF Models


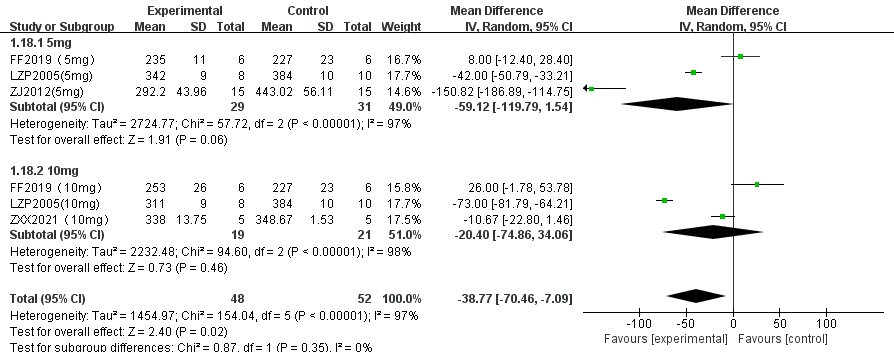


Fig. 25 Dosage Subgroup Analysis of Astragaloside IV on HR in Rodent MF Models

**Supplementary Materials 3: Subgroup Analyses by Cycle**

**
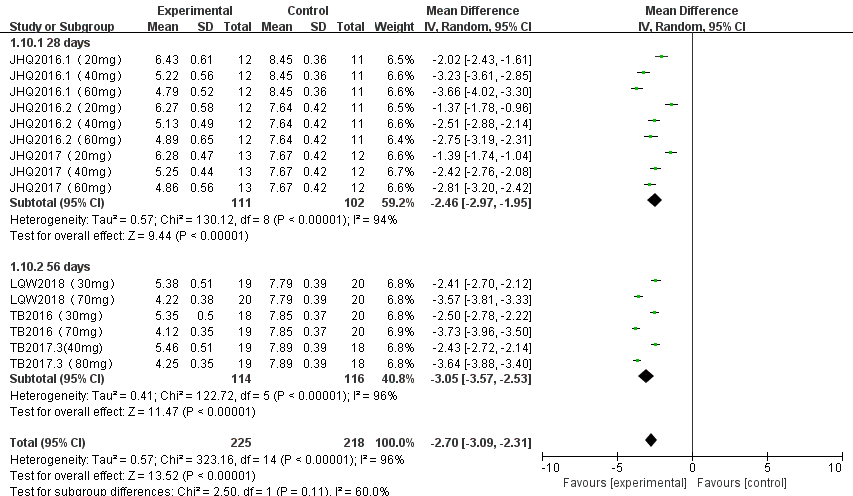
**

Fig. 26 Cycle Subgroup Analysis of Astragaloside IV on CVF in Rodent MF Models


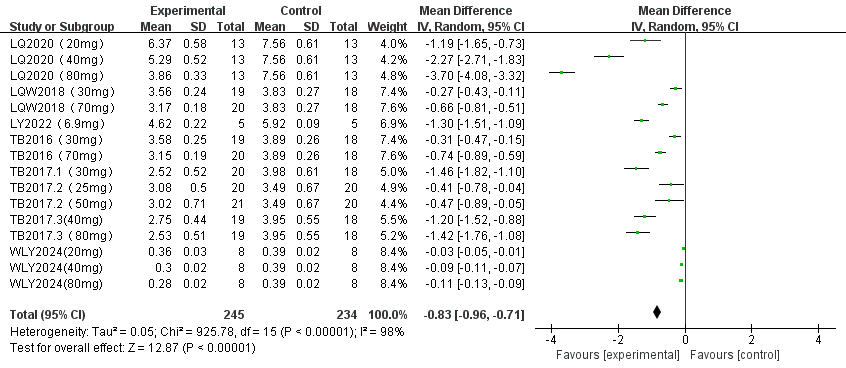


Fig. 27 Cycle Subgroup Analysis of Astragaloside IV on LVESd in Rodent MF Models


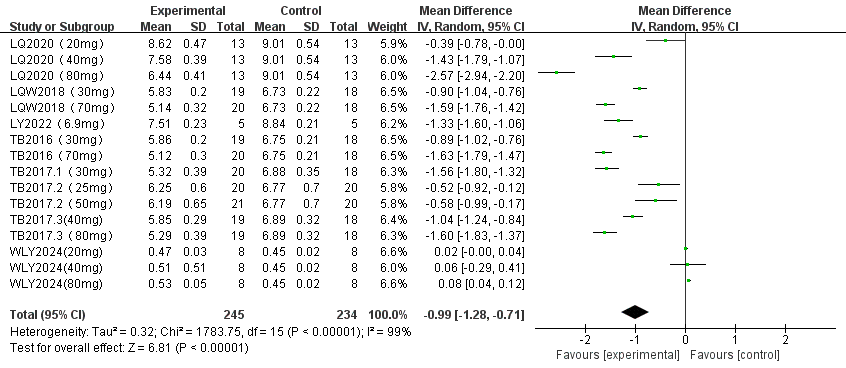


Fig. 28 Cycle Subgroup Analysis of Astragaloside IV on LVEDd in Rodent MF Models


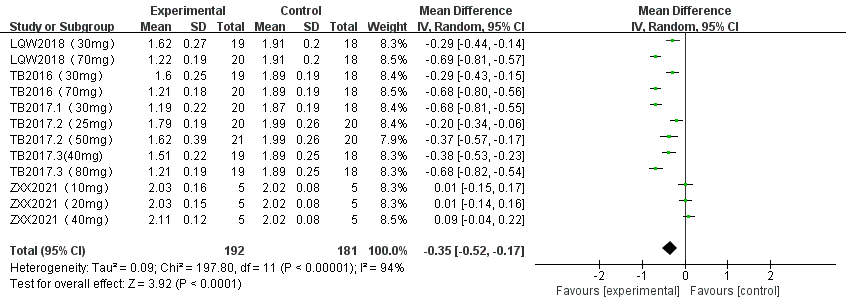


Fig. 29 Cycle Subgroup Analysis of Astragaloside IV on LVPWd in Rodent MF Models


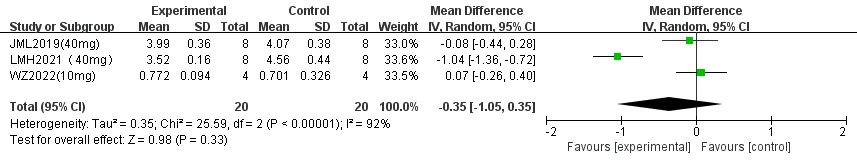


Fig. 30 Cycle Subgroup Analysis of Astragaloside IV on LVIDd in Rodent MF Models


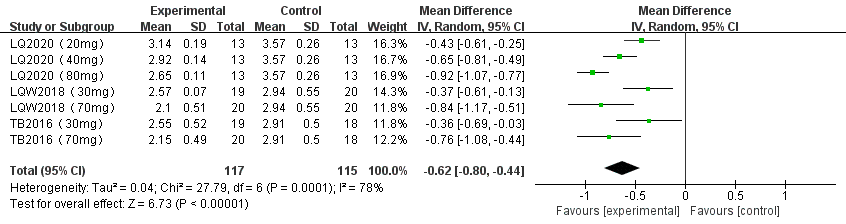


Fig. 31 Cycle Subgroup Analysis of Astragaloside IV on LVMI in Rodent MF Models


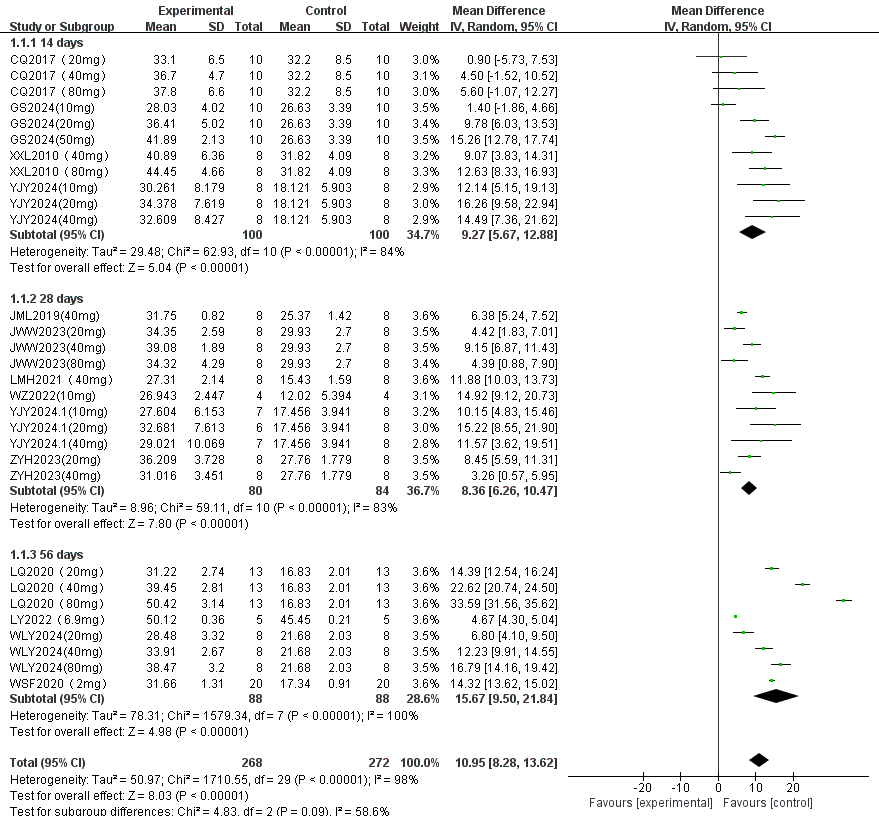


Fig. 32 Cycle Subgroup Analysis of Astragaloside IV on LVFS in Rodent MF Models


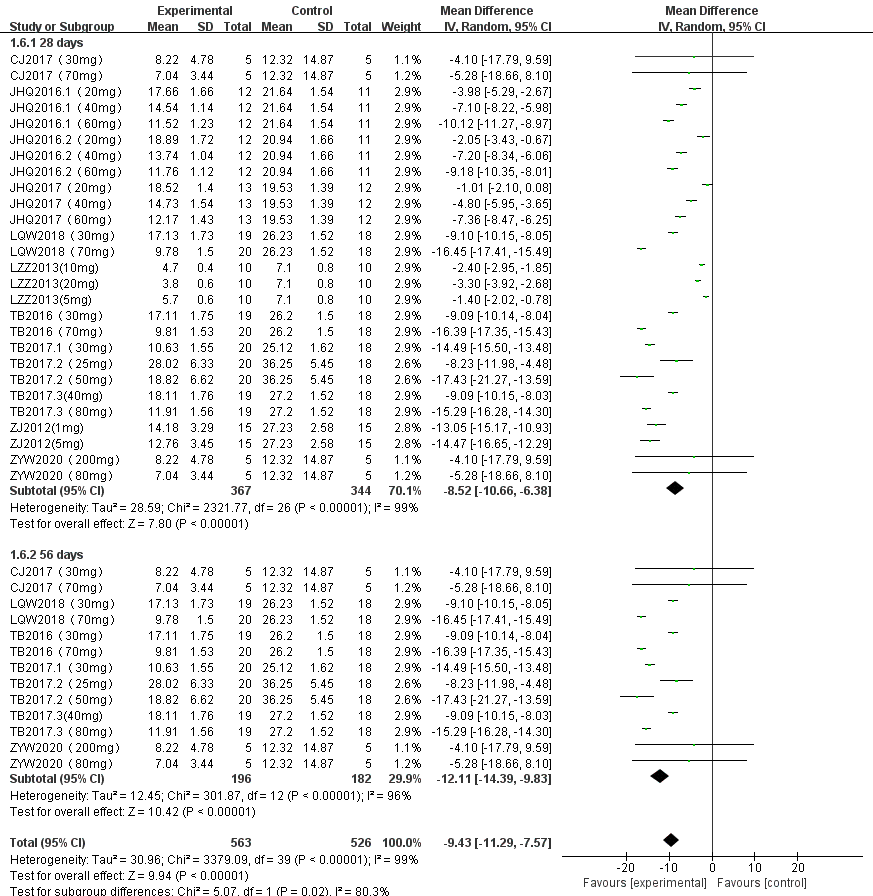


Fig. 33 Cycle Subgroup Analysis of Astragaloside IV on LVEDp in Rodent MF Models


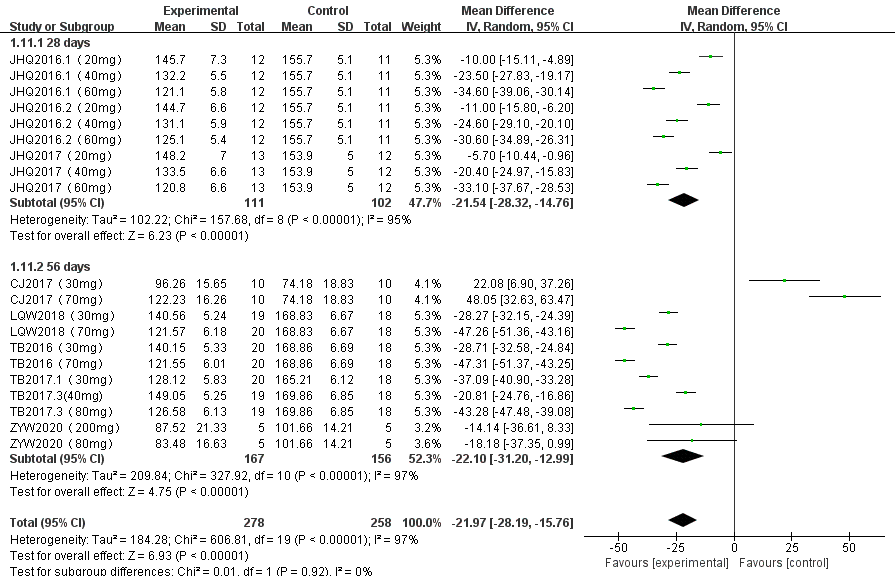


Fig. 34 Cycle Subgroup Analysis of Astragaloside IV on LVSP in Rodent MF Models


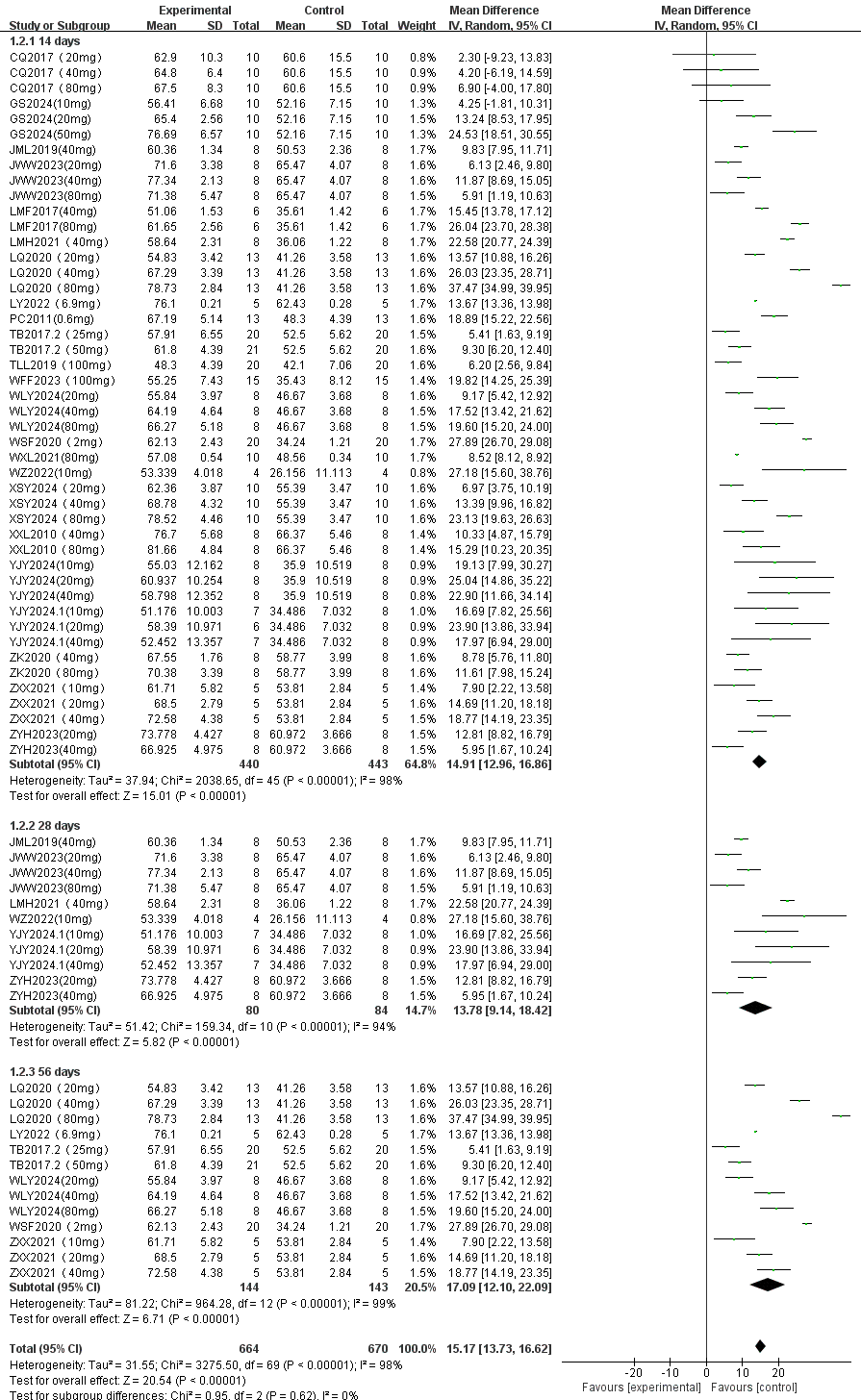


Fig. 35 Cycle Subgroup Analysis of Astragaloside IV on LVEF in Rodent MF Models


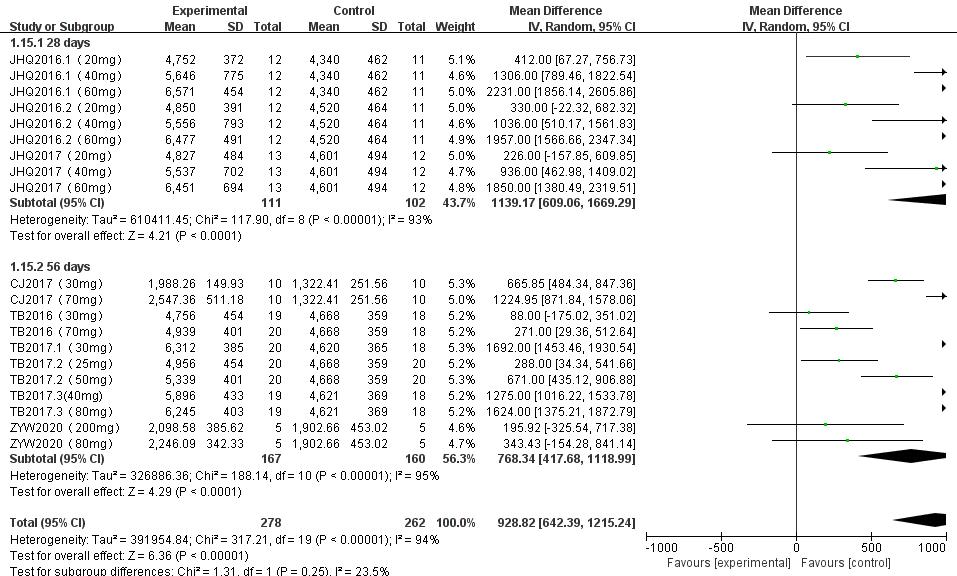


Fig. 36 Cycle Subgroup Analysis of Astragaloside IV on +dp/dtmax in Rodent MF Models


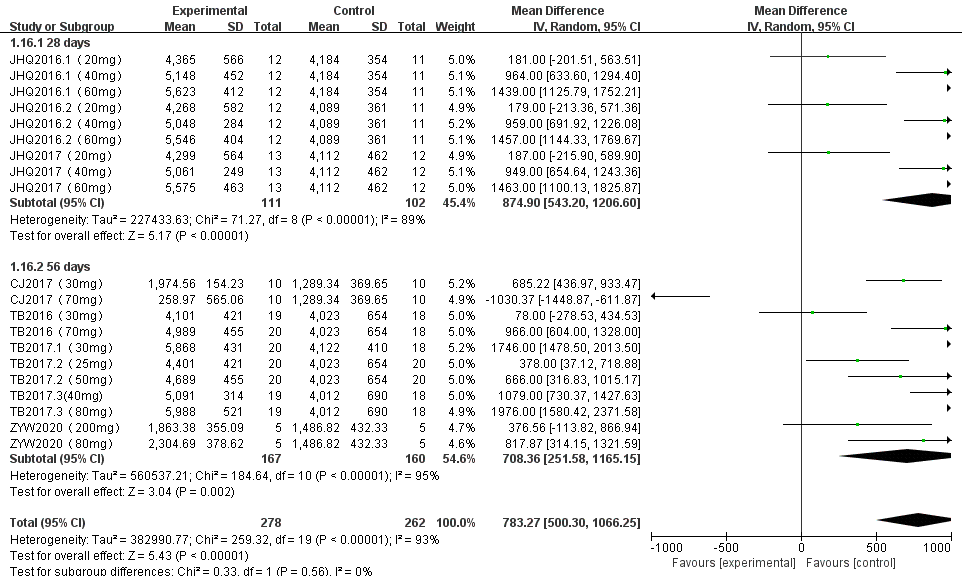


Fig. 37 Cycle Subgroup Analysis of Astragaloside IV on –dp/dtmax in Rodent MF Models


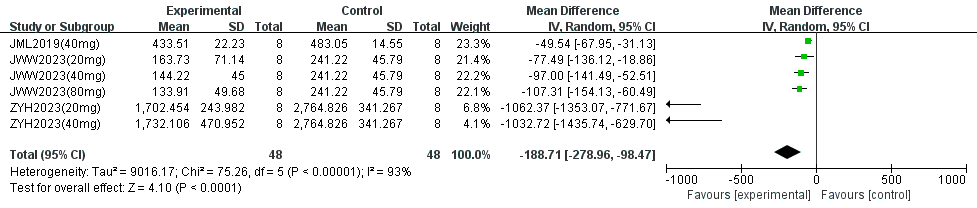


Fig. 38 Cycle Subgroup Analysis of Astragaloside IV on LDH in Rodent MF Models
