# Supplementary material for: The interventional effect of astragaloside IV on rodent models of myocardial fibrosis: a systematic review and meta-analysis
Source: Front Pharmacol. 2025 Sep 22;16:1625774. doi: 10.3389/fphar.2025.1625774 (PMC12497706; doi:10.3389/fphar.2025.1625774)
Supplement: Supplementary file 3 [file Supplementaryfile1.doc]

**Supplementary Materials 1: Search Strategies**

**CNKI**

| # | Searches |
| --- | --- |
| #1 | 全文=中英文扩展（心肌纤维化） |
| #2 | 全文=中英文扩展（心肌钙化） |
| #3 | 全文=中英文扩展（心脏纤维化） |
| #4 | 全文=中英文扩展（心内膜纤维化症） |
| #5 | 全文=中英文扩展（MF） |
| #6 | OR/1-5 |
| #7 | 主题=中英文扩展（黄芪甲苷） |
| #8 | 主题=中英文扩展（黄芪甲苷衍生物） |
| #9 | 主题=中英文扩展（AS-Ⅳ） |
| #10 | OR/7-9 |
| #11 | 主题=中英文扩展（大鼠） |
| #12 | 主题=中英文扩展（小鼠） |
| #13 | OR/11-12 |
| #14 | #6 AND #10 AND #13 |

**Wangfang**

| **#** | Searches |
| --- | --- |
| **1** | 主题:（心肌纤维化 或 心脏纤维化 或 心肌钙化 或 心内膜纤维化 或 MF） |
| **2** | 主题:（黄芪甲苷 或 黄芪甲苷衍生物 或 AS-Ⅳ） |
| **3** | 主题:（随机对照实验 或 RCT） |
| **4** | **1 AND 2 AND 3** |

**VIP**

| # | Searches |
| --- | --- |
| 1 | M=（心肌纤维化 或 心脏纤维化 或 心肌钙化 或 心内膜纤维化 或 MF） |
| 2 | U=（心肌纤维化 或 心脏纤维化 或 心肌钙化 或 心内膜纤维化 或 MF） |
| 3 | OR/1-2 |
| 4 | M=（黄芪甲苷 或 黄芪甲苷衍生物 或 AS-Ⅳ） |
| 5 | R=（黄芪甲苷 或 黄芪甲苷衍生物 或 AS-Ⅳ） |
| 6 | 0R/4-5 |
| 7 | M=（大鼠 或 小鼠） |
| 8 | T=（大鼠 或 小鼠） |
| 9 | OR/7-9 |
| 10 | 3 AND 6 AND 9 |

**Web of Science**

| **#** | Searches |
| --- | --- |
| **1** | TS=((((((Myocardial fibrosis) OR Myocardial calcification) OR cardiac fibrosis) OR cardiac muscle fibrosis) OR heart muscle fibrosis) OR Myocardial Interstitial Fibrosis) OR Cardiac fibrosis in myocardial) AND "Preprint Citation Index" (Exclude – Database) |
| **2** | TS=((Astragaloside OR Astragaloside IV) OR Astragaloside derivatives) AND "Preprint Citation Index" (Exclude – Database) |
| **3** | #1 AND #2 |
| **4** | TS=(randomized controlled trial OR RCT OR random controlled) |
| **5** | #3 AND #4 |

**Pubmed**

| **#** | Searches |
| --- | --- |
| **1** | (Myocardial calcification[MeSH Terms]) OR (Myocardial fibrosis[MeSH Terms]) |
| **2** | ((((cardiac fibrosis[Title/Abstract]) OR (cardiac muscle fibrosis[Title/Abstract])) OR (heart muscle fibrosis[Title/Abstract])) OR (Myocardial Interstitial Fibrosis[Title/Abstract])) OR (Cardiac fibrosis in myocardial[Title/Abstract]) |
| **3** | #1 OR #2 |
| **4** | ((Astragaloside[Title/Abstract]) OR (Astragaloside IV[Title/Abstract])) OR (Astragaloside derivatives[Title/Abstract]) |
| **5** | "Randomized Controlled Trials"[MeSH Terms] OR randomized controlled trial[Title/Abstract] |
| **6** | #3 AND #4 AND #5 |

**Embase**

| **#** | Searches |
| --- | --- |
| **1** | myocardial AND calcification OR (myocardial AND fibrosis) OR (cardiac AND fibrosis) OR (cardiac AND muscle AND fibrosis) OR (myocardial AND interstitial AND fibrosis) |
| **2** | ‘astragaloside’/exp OR astragaloside OR ‘astragaloside iv’/exp OR ‘astragaloside iv’ |
| **3** | 'randomized controlled trial'/exp OR randomized controlled trial.ab,ti |
| **4** | #1 AND #2 AND #3 |

**Cochrane Library**

| **#** | Searches |
| --- | --- |
| 1 | “Astragaloside IV” OR “AS-IV” OR “astragaloside” |
| **2** | “myocardial fibrosis” OR “cardiac fibrosis” OR “myocardial calcification” OR “cardiac muscle fibrosis” OR “myocardial interstitial fibrosis” |
| **3** | #1 AND #2 |
| **4** | "randomized controlled trial OR "RCT" |
| **5** | #3 AND #4 |
